# Recovery of soil microbiota in naturally regenerating *Acacia mangium* ecosystems

**DOI:** 10.7717/peerj.21048

**Published:** 2026-04-13

**Authors:** Jenny Vivian, Michelle Scriver, Anastasija Zaiko, Alison Shapcott, Robin L. Chazdon, Alexandra A. Catling, David J. Lee

**Affiliations:** 1 Forest Research Institute, University of the Sunshine Coast, Sippy Downs, Queensland, Australia; 2 Sequench Ltd, Nelson, New Zealand; 3 School of Science, Technology and Engineering, University of the Sunshine Coast, Sippy Downs, Queensland, Australia; 4 Centre for Bioinnovation, University of the Sunshine Coast, Sippy Downs, Queensland, Australia

**Keywords:** Soil microbial communities, eDNA, High-throughput sequencing, Forest restoration, *Acacia mangium*, Soil fungi, Soil bacteria, Forest natural regeneration, Tropical forest, Tropical soil microbes

## Abstract

Forest ecosystem restoration often focuses on the recovery of tree diversity and carbon stocks, with minimal attention given to soil microbial communities. Given the essential role of soil microorganisms for ecosystem health and recovery, this lack of understanding may limit reforestation success. In this study, we address this gap by analysing the taxonomic and functional characteristics of fungal and bacterial communities in minimally managed *Acacia mangium* plantations, especially considering their role in carbon sequestration and ecosystem functioning. We sampled naturally regenerating *Acacia mangium* plantations aged 2, 10, and 24 years, grasslands of *Imperata cylindrica* as the baseline condition, and remnant forests as reference state. We identified soil fungal and bacterial taxa through high-throughput amplicon sequencing of soil environmental DNA (eDNA), targeting ITS and 16S rRNA metabarcoding markers. Our results showed that microbial community and functional groups composition differed among landcover types, although taxonomic richness did not. Bulk topsoil organic carbon, pH, and total nitrogen were key factors associated with the composition of microbial communities, especially dominant fungal phyla. Symbiotrophic fungi and copiotrophic bacteria generally increased and recovered over time, potentially enhancing carbon (C) sequestration and balancing nutrient cycles. These findings demonstrate that natural regeneration in *A. mangium* plantations can promote the recovery of soil microbial communities and their associated functions, including those relevant for climate change mitigation. Furthermore, our study highlights the effectiveness of eDNA and high-throughput sequencing in monitoring early ecosystem shifts in soil microbial communities, which could be used to guide reforestation efforts towards desired ecosystem services.

## Introduction

Tropical forest destruction due to anthropogenic activities is an increasing concern intensified by climatic changes due to the accumulation of atmospheric greenhouse gases (Stott, Stone & Allen, 2004; Field et al., 2012). Deforestation is threatening the survival of tropical forests (Myers, 1993), hampering biodiversity and reducing ecosystem services such as carbon (C) sequestration (Hoang & Kanemoto, 2021). In this context, conservation and reforestation are critical nature-based solutions to mitigate negative climatic changes (Griscom et al., 2017) and improve ecosystem health. The value of such approaches relies on the ability of tropical forests to provide persistent above- and below-ground C sinks (Pan et al., 2011).

Despite the use of multiple species being recommended for reforestation practices (Liu, Kuchma & Krutovsky, 2018), monocultures were often established by forestry agencies because of economic considerations and ease of management. In this context, *Acacia mangium* became one of the leading tree species used in tropical regions for reforestation plantings, with its fast-growing traits (Potter, Rimbawanto & Beadle, 2006), and exotic framework species characteristics, such as the ability to promote forest recovery (Elliott et al., 2023). Generally, *A. mangium* plantations were only managed for three years after planting ended, due to social and economic constraints (Chokkalingam et al., 2006; Gregorio et al., 2015), leading to a lack of long-term management and environmental monitoring. Over the last decades, numerous efforts have been made to reforest degraded lands, with multiple key drivers of successful reforestation (*e.g.*, site characteristics and indicators of forest growth; Le et al., 2012; Le, Smith & Herbohn, 2014), being poorly or only briefly monitored, and therefore hampering the demonstration of the achievement of their goals (Bash & Ryan, 2002; Zhao, 2023). To enable better monitoring of reforested areas, the combination of different metrics (*e.g.*, vegetation structure, litter production, species diversity) has been proposed (Aronson et al., 1993; Hobbs & Norton, 1996; Ruiz-Jaén & Aide, 2005). However, examinations of soil microbial communities are rare (Pedrinho et al., 2024; Yan et al., 2018), despite their central role in ecosystem processes linking biotic and abiotic conditions (Lemke & De Salle, 2023; McCulloch et al., 2024). This gap often arises from the limitations of traditional microbe-based approaches, being time-consuming, requiring specialised culturing techniques and high levels of expertise to identify fungal and bacterial taxa (Franco-Duarte et al., 2019). Furthermore, given the large number of unique microbial taxa present in soil, the traditional approach is inefficient and leads to a lack of uptake in forestry land management (Vivian et al., 2025). Nevertheless, recent advancements in high-throughput sequencing and the expanding field of environmental DNA (eDNA) (genetic material collected from environmental substrates; Thomsen & Willerslev, 2015) provide a time and cost-efficient evaluation of soil microbes and their functions, which stand at the basis of ecosystem health.

Soil fungi and bacteria processes are intertwined with ecosystem functioning, making them useful indicators of environmental modifications and C sequestration potential (Bardgett & Van Der Putten, 2014). Microbial organisms thriving in the soil influence local vegetation growth and ecosystem stability (Zak et al., 2003), as they play an important role in organic matter decomposition and soil organic carbon (SOC) turnover (Bardgett & Van Der Putten, 2014). Because microbes are physically attached to their substrate (Yan et al., 2018), they can promptly respond to variations in microclimatic conditions (Vasconcellos et al., 2014; Xue et al., 2016), making them susceptible to short- and long-term ecosystem changes (Bardgett & Van Der Putten, 2014; Li, Shangguan & Deng, 2020), and therefore early indicators of restoration success (Harris, 2003; Ma et al., 2022; Graham & Knelman, 2023).

The taxonomic and functional profile of soil microbial communities can elicit a better understanding of forest recovery and C sequestration processes. Taxonomic variation of bacterial and fungal communities tracked vegetation restoration trajectories in different forest biomes, including tropical forests (*e.g.*, Gellie et al., 2017; Adamo et al., 2021). Related to their functions, the transition from a microbial community dominated by oligotrophic bacteria (*i.e.,* adapted to a nutrient-poor environment) and saprotrophic fungi to one rich in copiotrophic (*i.e.,* thriving in nutrient-rich conditions) and symbiotrophic organisms marked the recovery of the forest to historical conditions and high CO_2_ sequestration potential (*e.g.*, Gehring et al., 2005; Batterman et al., 2013; Gei et al., 2018; Taylor, Chazdon & Menge, 2019; Wang et al., 2020; Liu et al., 2022).

Our study addresses the lack of investigations into the modification of taxonomic and functional traits of soil microbial communities in tropical forest restoration within *A. mangium* plantations. The study was undertaken in the Philippines, where multiple reforestation projects were implemented at different times over the last 30 years (Gregorio et al., 2015), creating a matrix of minimally managed and naturally regenerating *A. mangium* plantations, which started as monocultures. To the best of our knowledge, only one previous study has compared the microbial community of *A. mangium* plantations (managed according to the “natural restoration” concept) to native mixed forests, in subtropical China (Wei et al., 2024). Thus, further research is needed on soil microbial variation in an *A. mangium* reforestation context (Koutika & Richardson, 2019). To unravel the parallel modification of soil microbial communities, forest recovery, and their influence on C sequestration, we analysed eDNA retrieved from bulk topsoil samples, hypothesising an increasing resemblance of the soil microbial community taxonomic and functional traits over time to those of the historical state of the remnant forest. Therefore, we addressed the following questions: (i) What is the taxonomic diversity of fungal and bacterial communities in *A. mangium* plantations of different ages, compared to reference states? (ii) How does the community composition of soil fungi and bacteria change as natural regeneration proceeds in *A. mangium* plantations? (iii) How do functional traits of fungal and bacterial communities vary with forest recovery in naturally regenerating *A. mangium* plantations? (iv) What are the implications of fungal and bacterial community variations for C sequestration and forest restoration assessment?

By answering these questions, our analysis provides the groundwork for a better understanding of the characteristics of soil fungal and bacterial communities in a widespread type of plantation, with influences on the C sequestration and related global effects.

## Methodology

### Study site

**Figure 1:**
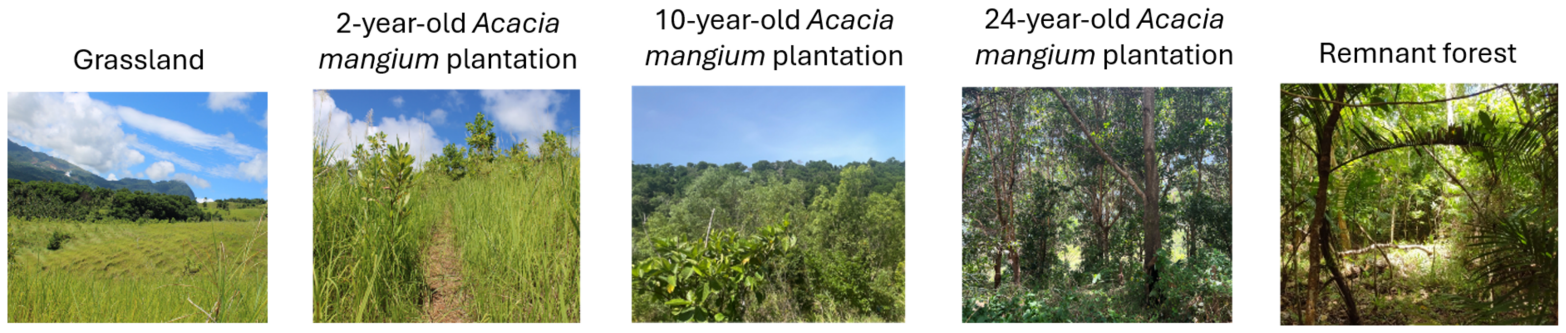
Chronosequence studied. Study sites sampled for investigating the recovery of the microbial community during reforestation using *A. mangium*.

To reduce secondary environmental effects on results, all sites were sampled within the same study area located in the Province of Biliran, Philippines (Fig. S1). The climate is tropical and monsoonal. While not distinct, the wet season occurs from May to October and the dry season from November to April. Evergreen tropical rainforest with a closed canopy of dipterocarps is the native vegetation on the coastal hills and uplands of this region (Peque & Hölscher, 2014). Deforestation has been extensive throughout the past century, and most of the primary forests have been replaced with coconut plantations, agricultural land, or *Imperata cylindrica* grasslands (Le, Smith & Herbohn, 2015). The study sites included a 2-year-old *A. mangium* plantation (part of Project Tarsier, a community-based reforestation project for carbon initiative), 10-year-old *A. mangium* plantation (established through the Australian Centre for International Agricultural Research Watershed Rehabilitation Project; Gregorio et al., 2015), and 24-year-old *A. mangium* plantation (part of a precedent project awarded to the local People Organizations by the Philippines Department of Environment and Natural Resources) (Fig. 1). Grasslands dominated by *Imperata cylindrica* and *Saccharum* spp. in former forest ecosystems were selected (considering their land-management history and proximity to the other sampled sites) as the initial condition before the establishment of *A. mangium* plantations. As a reference for the goal ecosystem state, a local remnant forest was sampled (Fig. 1). This area is composed of historical primary and secondary forests, anthropogenically not extensively disturbed since the end of the colonial era around 1950 (Emtage, 2004; Suarez & Sajise, 2010). The remnant forest has been part of the Key Biodiversity Area since 2006 (Key Biodiversity Areas Partnership, 2025; BirdLife International, 2025), and it fits the purpose of a reference for the target restoration state due to high biodiversity and current low anthropogenic disturbance. The three plantations and the two reference states are hereafter referred to as “landcover types”.

Within each landcover type, we established four transects along an altitudinal gradient, each of them containing four 5 × 5 m plots spaced 10 m apart, for a total of 16 plots (Fig. S2). The altitudinal effect was minimised by disposing pairs of two sets of transects at the same elevation range, with one set facing North and the other South to consider the different light exposure (Fig. S3). Overall, the plots are spread across a 1,800 m wide area, with a maximum elevation difference of 400 m between plots and are located over basaltic deposits on one side of the Asluman inactive volcano (Mendoza, Balangue-Tarriela & Reed, 2019). Abiotic characteristics of the sampled sites are summarised in Table S1. A buffer area (at least 10 m or 2.5 times the height of the trees located at the landcover edges) was included for each sampling site to avoid the influence of edges with zones in which other projects were implemented. All the selected sites were potentially impacted in 2013 and 2019 by Super Typhoons Yolanda and Orduha. Sample collection occurred in September 2024, across a total of 80 plots (*i.e.,* 16 plots for each of the five landcover types; Fig. S4). The recording of the data and soil samples collection were authorized by the local government agency as part of research activities under Project Tarsier.

### Soil properties

Soil properties were measured at each sampling location (*i.e.,* in 16 plots for each landcover type) as part of a broader study, with methods described by Vivian et al. (2026). Briefly, soil pH, temperature, and moisture were measured with a multi-purpose probe (Mcbazel, model MT-gf-020598). Additionally, a subsample of soil of 100 g (0–10 cm depth) was collected from the centre of each plot after mixing the surface layer and retained in zipper-sealed bags. The soil samples were kept in a cooler bag to slow microbial activity and related effects on the chemical characteristics of the substrate until arrival in storage facilities, where they were placed in fridges (at 1–4 °C). Then, the samples were transferred to the Visayas State University laboratory, where the chemical analyses were conducted. These evaluations determined the SOC content following the protocol of Heanes (1984); the total nitrogen (TN) through steam distillation method (Bremner & Keeney, 1965), and available phosphorus (AP) utilizing the Olsen procedure (Olsen, 1954).

### Molecular methods

#### Sampling

Soil samples for molecular analysis consisted of approximately 1 g of mixed topsoil (0–10 cm) from the centre of each 5 × 5 m plot. This subsample was placed in two mL sterile Eppendorf tubes containing one mL of the DNA/RNA Shield™ buffer (Zymo Research, USA) for DNA preservation. The usage of 1 g (or less) per soil sample is in line with standard procedures and kits (*e.g.*, Bruce et al., 2021), and common practice in other studies (*e.g.*, extraction of 0.25 g of dry soil per sample; Fernandez Nuñez et al., 2021; Manici et al., 2024). To ensure sterility and consistency, only one researcher conducted the soil sampling, being the first person to set up and be present in the established plots. Fresh gloves and sterilisation of the equipment with ethanol (spatula for soil collection) were undertaken before the collection of samples from each plot. Duplicate samples were gathered at each of the 80 sampling points described above, totalling 160 samples. For each transect pair (south and north side of the ridge), we collected duplicated field controls in the lowest elevation plots in the transect on the south side of the ridge (totalling 20 field controls). The field blanks (10 pairs, each gathered on the lowest plot of the Southern facing transect) were prepared, handled, and processed the same way as the biological soil samples, but without soil collection, and used to evaluate potential contamination during field operations. Each sample vial was sealed with parafilm and stored in zipper-sealed bags. For each site, samples were collected and stored in insulated bags until nearby storage at 1–4 °C. One complete set of vials (80 samples and 10 blanks) was sent to Sequench Ltd (Nelson, Aotearoa, New Zealand) for sequencing and stored at 4 °C until processed.

#### Environmental DNA extraction and high-throughput sequencing

One complete set of soil samples (*n* = 90, with 80 soil samples and 10 field blanks) was analysed at Sequench Ltd (Nelson, Aotearoa, New Zealand) between November and December 2024, sequencing eDNA fragments of fungi and bacteria identified through metabarcoding. The DNA extractions, amplicon PCR, and library preparation were conducted in separate sterile laboratory rooms to avoid cross-contamination. The sterilization of each room was ensured by treating the air in each area with UV light for a minimum of 30 min before each use. The amplicon PCR was undertaken in a DNA/RNA UV-cleaner box with a UV cleaner–recirculator (BioSan, Riga, Latvia). Aerosol barrier tips (Accumax, Interlab, Wellington, NZ) were used throughout sample processing.

DNA was extracted from soil samples using the ZymoBIOMICS MagBead DNA/RNA (Zymo, Irvine, CA, USA) kit on the KingFisher Flex (Thermo Fisher, Waltham, MA, USA) system. All samples were homogenized in ZR BashingBead Lysis Tubes with 750 µL of DNA/RNA Shield and by bead beating for 20 min using the MPS-1 Multi-Plate Shaker (BioSan, Riga, Latvia) at 1800 RPM. Afterwards, they were centrifuged at 13,500 RPM for 5 min at 20 °C with a High-Speed Micro CF-10 Centrifuge (Daihan Scientific, South Korea). DNA was extracted from each sample following the reference guide of the manufacturer for the KingFisher Flex. Negative controls (DNA extraction blanks) were included in each extraction run.

Metabarcoding analysis was performed using two primer sets for fungi and bacteria: the internal transcribed spacer 2 (ITS2) region of the nuclear ribosomal RNA (rRNA) gene for fungi (White et al., 1990), and the V3–V4 region of the nuclear small-subunit ribosomal RNA (16S rRNA) gene for bacteria (Klindworth et al., 2013). Following the guidelines of Kozich et al. (2013), primers used with Illumina overhang adaptors were: ITS3_Illumina_tag 5′-GCATCGATGAAGAACGCAGC-3′, and ITS4_Illumina_tag 5′-TCCTCCGCTTATTGATATGC-3′ for fungi; NexR-16SFWD 5′-CCTACGGGNGGCWGCAG-3′, and NexR-16SREV 5′-GACTACHVGGGTATCTAATCC-3′ for bacteria (for forward and reverse transcription, respectively). Amplifications of the target gene regions were conducted using a T100 Thermal Cycler (Bio-Rad, Hercules, CA, USA) with 15 µL of MyFi Mix (Bioline, London, United Kingdom), 1.5 µL for fungi and 0.75 µL for bacteria of each 10 µM primer, 9 µL for fungi and 11 µL for bacteria of DNA-free water, and 3 µL of template DNA, in a total volume of 30 µL. For fungi eDNA amplification, the thermocycling conditions were 94 °C for 5 min, followed by 35 cycles of 94 °C for 30 s, 55 °C for 30 s, 72 °C for 40 s, and a final extension of 72 °C for 10 min (Park et al., 2023). For bacteria, thermocycling conditions were 95 °C for 1 min, followed by 35 cycles of 95 °C for 15 s, 50 °C for 15 s, 72 °C for 15 s, and a final extension of 72 °C for 7 min. Negative (no template) PCR controls were included in all PCR runs. Amplification products of the 16S rRNA and ITS2 genes were purified and normalized using the SequalPrep Normalization kit (Thermo Fisher, Waltham, MA, USA), resulting in a concentration of ∼1 ng/µL.

Cleaned amplicons were indexed with the xGen™ Amplicon UDI Primers (Integrated DNA Technologies, Newark, NJ, USA), and high-throughput sequencing was performed on the Illumina NextSeq 2000 platform. During library preparation, a water blank (*i.e.,* sequencing control) was included to track potential contamination at this step. The library pool, diluted to a final loading concentration of 600 pM with a 25% PhiX spike, was sequenced using NextSeq Reagent X-LEAP kit P1-600 cycle (2  × 301 bp) (Illumina, San Diego, CA, USA).

#### Sequence processing

To assign taxonomic information on the retrieved sequences, we used the Amplicon Sequence Variants (ASVs), an approach commonly adopted in applied environmental sequencing (*e.g.*, Forster et al., 2019; Fernandez Nuñez et al., 2021; Giebner et al., 2020). Compared to the usage of Operational Taxonomic Units (OTUs), ASVs allow a higher distinction of sequences (Callahan, McMurdie & Holmes, 2017; Chiarello et al., 2022).

Similar bioinformatic pipelines were used to assign ASVs for ITS2 and 16S rRNA sequences. The bioinformatic pipeline was run as follows. Cutadapt v5.0 (Martin, 2011) was used to remove the primer sequences from the raw reads, applying an error rate of 0.1 for primer mismatches. After this, sequences were processed using the “DADA2” package (Callahan et al., 2016) in the R environment (R Core Team, 2024). Reads were truncated to 226 and 220 bp for 16S rRNA forward and reverse reads. Conversely, ITS2 sequences were not truncated, given the known length variation at this locus (Kyle & Klassen, 2025). Both ITS2 and 16S rRNA were filtered with a default maximum number of “expected errors” (maxEE) of 2. Then, a parametric error matrix was constructed, forward and reverse sequences were dereplicated and denoised, during which most low-abundance singletons were filtered. Paired-end reads were merged with no mismatches allowed, and a minimum overlap of 10 bp. Chimeric sequences were filtered out by the consensus option in ‘removeBimeraDenovo’ function. For the taxonomic classification of ITS2 rRNA gene amplicons, we used the UNITE database (version 10.0) (Abarenkov et al., 2024), while for the 16S rRNA gene amplicons, ASVs were matched against the SILVA database (version 138.2) (Quast et al., 2013). Taxonomic assignment was performed using the “DADA2” ‘assignTaxonomy’ script and the RDP classifier (Wang et al., 2007), with default settings. Given the lack of microbial inoculation in all the plantations considered, the maximum sequence found in controls (field, extraction, PCR, and sequencing blanks) was used as a threshold for contaminating ASVs (Bell et al., 2019). Thus, the ASVs detected in the environmental samples and controls with fewer reads than the threshold were excluded from the analysis using the “ps_decon” function from biohelper v0.0.16.000 (https://github.com/olar785/biohelper) with the “microdecon” method (McKnight et al., 2019), selecting the max_v method. For environmental samples, each ASVs exceeding the threshold had the threshold value subtracted from the sequence count to mitigate contamination effects.

Numerous fungal and bacterial taxa are yet to be identified, and their genome completely sequenced (Beng & Corlett, 2020). Therefore, despite using updated genetic libraries to assign the detected sequences to unique ASVs, a portion of them were not aligned to known taxa or functional groups (these being based on genetic information). In our study, these ASVs were classified as “unknown”. Considering both the identified and “unknown” ASVs, we analysed the composition and taxonomic and functional characteristics of the microbial communities, at different taxonomic levels (phylum, genus, and ASV), and for subsequent analyses.

### Statistical analyses

Data were analysed at the landcover-level, treating each 1 g from every plot as a replicate, capturing the heterogeneity in soil communities and taxa. We considered the variability between the communities within the landcover types (*i.e.,* between transects) to discuss the significant differences among plantation stages. To evaluate sequencing depth adequacy and standardize the read number across samples for alpha and beta diversity calculation, rarefaction curves were created for both fungi and bacteria (Figs. S5 and S6) using the “vegan” v2.6-8 package (Oksanen et al., 2024). This approach allowed the determination of a threshold that balanced sample retention and taxonomic coverage and controlled for uneven sampling effort (Schloss, 2024). The rarefaction threshold was selected based on rarefaction curves (the point at which the observed richness for each sample reached a plateau) and the ordered number of total reads per sample, using the function “sample_sums” in the “phyloseq” v1.42.0 package (McMurdie & Holmes, 2013). For fungal sequences, data were rarefied to 24,803 reads, resulting in the removal of two samples (the first two from the initial transect in the remnant forest). For bacterial sequences, data were rarefied to 34,956 reads, and four samples were discarded (one 10-year-old plantation, two samples from the 24-year-old plantation, and one from the remnant forest) as a result. Therefore, the 10-year-old plantation and remnant forest held a total of 15 replicates, the 24-year-old plantation 14, the 2-year-old plantation, and the grassland 16.

Using the packages “vegan” v2.6-8 (Oksanen et al., 2024), and “microeco” v1.10.0 with its dependencies (Liu et al., 2021), fungal and bacterial taxa relative abundances (based on the number of sequences belonging to each ASVs) and ASVs richness were estimated, based on the sampled and rarefied data. Data normality was assessed using the Shapiro–Wilk test, after which Kruskal–Wallis tests followed by *post hoc* Dunn’s test were applied to identify significant differences in abundances and alpha diversity metrics (Shannon–Weaver index, and observed ASVs) between and within landcover types (grassland, 2-, 10-, and 24-year-old plantations, and remnant forest). We also reported the exponential Shannon-Weaver index (Hill number of order *q* = 1) to more intuitively show the effective number of species between landcover types (Chao et al., 2014). This latest index of diversity was derived by taking the base of the natural logarithm (*e*) to the power of the previously calculated Shannon–Weaver value (Chao et al., 2014), for each sample. Average and standard deviations were calculated using “psych” v2.5.6 (Revelle, 2025), and the Kruskal–Wallis test, followed by *post hoc* Dunn’s test, were applied to assess significant differences among landcover types.

The tests for community similarities were run separately for fungi and bacteria. For this analysis, the Jaccard distance metric based on incidence was considered, given its advantages compared to other distance metrics (see Chao et al., 2005; Chao et al., 2006). Permutational multivariate analysis of variance (PERMANOVA) was used to assess the difference between microbial communities of different landcover types. The interpretation of the PERMANOVA results was corroborated by the calculation of the dispersion within groups through the PERMDISP test (Anderson et al., 2011), recognizing whether the significant difference in the taxonomic composition could have been prompted by a different dispersion value rather than a true difference in community structure. Principal Coordinates Analysis (PCoA) was used to visualize the similarities between communities. We applied the linear discriminant analysis (LDA) effect size (LefSe) to identify microbial phyla and genera that were differentially abundant between landcover types. The LDA score indicates the taxa driving differences between communities, allowing identification of taxa characterizing the differently aged *A. mangium* plantations and reference sites (Segata et al., 2011). Thresholds for the most discriminant groups were set as follows: logarithmic LDA score ≥3.9 for fungal phyla, ≥3 for bacterial phyla, and ≥3 for bacterial genera, with *α* ≤ 0.01 in all cases (factorial Kruskal–Wallis test).

Distance-based Redundancy Analysis (db-RDA) and partial Mantel test (Smouse, Long & Sokal, 1986; Legendre & Legendre, 2012) were conducted to assess relationships between compositional dissimilarities and environmental factors, using the Pearson coefficient of correlation and the Jaccard metric for distances. The environmental factors considered were SOC (%), TN (%), AP (mg/kg), C:N, C:AP, air and soil temperature (°C), air and soil humidity (%), and soil pH. With the representation of beta diversity components as pairwise distance matrices, we implemented the common analytical tool of the Partial Mantel test to unravel the correlation of microbial groups with environmental parameters. For the interpretation of this analysis, a positive correlation between environmental parameters (as listed above) and the beta diversity of the microbial community indicates that the increase in the environmental parameter value is accompanied by an increase in beta diversity of the microbial community. The opposite applies to a negative correlation.

After the taxonomic assignment, the detected taxa were assigned to the functions they can express, through the “microeco” package v1.10.0 (Liu et al., 2021), using the FUNGuild database (Nguyen et al., 2016) for fungi and the FAPROTAX database v1.2.10 (Louca, Parfrey & Doebeli, 2016) for bacteria. Specifically, this method considers the classification of microbial taxa into functional groups given their genetic composition, related to previously assessed expression of specific metabolic processes and ecological traits. Differences among relative abundances of functional groups were tested using the Kruskal-Wallis and Dunn *post hoc* tests. Principal Component Analysis (PCA) was used to visualize the similarities between communities for their functional profile. Statistical analyses and plotting were performed in the R environment (R Core Team, 2024) using the package “ggplot2” v3.5.1 for visualisation (Wickham, 2016).

## Results

**Figure 2:**
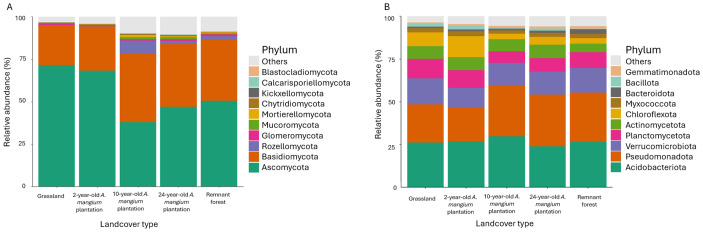
Relative abundances of the studied microbial communities. Relative abundance of the top ten fungal (A) and bacterial (B) phyla in each land cover type based on sequenced ASVs.

A total of 1,934,634 (12,006 unique ASVs) and 2,656,339 sequences (84,782 unique ASVs) were retained for fungi (ITS2) and bacteria (16S), respectively, after quality filtering and denoising. From these, 1,510 ASVs, 848 species, 968 genera, 446 families, 180 orders, 60 classes, and 13 phyla were identified for fungi, and 1,947 ASVs, 729 species, 1,290 genera, 582 families, 358 orders, 144 classes, and 51 phyla for bacteria (Figs. 2A–2B).

### Fungal and bacterial taxonomic diversity over natural regeneration in *A. mangium* plantations

#### ASVs alpha diversity between each landcover type

The number of observed ASVs (*i.e.,* richness) of fungi and bacteria was similar over the forest recovery timeline (Table 1). Shannon-Weaver index (standard and exponential) of fungi demonstrated comparable values among landcover types, as did the bacteria.

**Table 1:** Average alpha diversity represented by observed ASVs richness, Shannon-Weaver index, and exponential Shannon-Weaver index (*i.e.*, Hill number of order 1) with standard deviation across landcover. Kruskal–Wallis tests displayed no significant differences among diversity values.

	**Fungi**			**Bacteria**		
	**Indices of diversity**					
**Landcover types**	**Richness**	**Shannon-Weaver**	**Exponential** **Shannon-Weaver**	**Richness**	**Shannon-Weaver**	**Exponential** **Shannon-Weaver**
**Grassland**	246.9 ± 7.4	4.2 ± 0.3	69.1 ± 17.2	1,757.6 ± 454.3	6.5 ± 0.3	665.4 ± 161.0
**2-year-old plantation**	216.8 ± 95.1	4.1 ± 0.4	62.7 ± 18.2	1,785.2 ± 359.5	6.6 ± 0.2	783.4 ± 158.3
**10-year-old plantation**	231.9 ± 78.6	4.1 ± 0.4	61.4 ± 21.8	1,875.3 ± 332.6	6.6 ± 0.2	713.6 ± 134.4
**24-year-old plantation**	249.8 ± 141.1	4.2 ± 0.7	78.5 ± 47.6	1,757.9 ± 495.0	6.5 ± 0.3	719.5 ± 249.6
**Remnant forest**	324.0 ± 130.2	4.3 ± 0.6	85.4 ± 39.7	2,184.5 ± 691.6	6.8 ± 0.4	931.2 ± 402.2

#### Taxonomic profiles

Ascomycota (55 ± 16%) and Basidiomycota (32 ± 14%), Acidobacteriota (27 ± 5%) and Pseudomonadota (26 ± 6%) were the dominant fungal and bacterial phyla, respectively, across the landcover types (Figs. 2A–2B). The most abundant genera were *Tomentella* (5 ± 9%), *Trechispora* (2 ± 3%), and *Clavulinopsis* (2 ± 7%) for fungi, and *Candidatus Udaeobacter* (8 ± 4%), *Candidatus Solibacter* (4 ± 1%), and *Acidibacter* for bacteria (2 ± 1%).

The relative abundances of sequences belonging to different taxa varied across landcover types. For the fungal community, the Ascomycota phylum was significantly more abundant (*p* < 0.01) in the grassland compared to the other landcover types (Table 2, Fig. 2A). Basidiomycota, Rozellomycota, Mortierellomycota, Mucoromycota, Chytridiomycota, and Kickxellomycota phyla distinguished the 10-year-old plantation from all the other landcovers types (*e.g.*, Rozellomycota 8 ± 5% in the 10-year-old plantation, while 1 ± 2% in the other landcover types, Fig. 2A).

**Table 2:** Fungi and bacteria Linear Discriminant Analysis scores (while threshold of ≥ 3.9 for fungal phyla, ≥3 for bacterial phyla, and *p* < 0.01) for phyla characterising distinct landcover types taxa with lower LDA scores are here displayed to share additional information on taxa characterising (although to a lesser extent) the landcover.

Fungi			Bacteria		
Phyla	**Landcover**	**LDA**	**Phyla**	**Landcover**	**LDA**
Ascomycota	Grassland	5.2	Planctomycetota	Grassland	4.4
Basidiomycota	10-year-old plantation	4.9	Bacillota		3.9
Rozellomycota		4.6	Chloroflexota	2-year-old plantation	4.7
Mortierellomycota		4.0	Dependentiae		3.3
Chytridiomycota		4.0	FCPU426		2.9
Mucoromycota		3.9	Elusimicrobiota	10-year-old plantation	2.9
			Candidatus Eremiobacterota		3.7
			Pseudomonadota	24-year-old plantation	4.7
			Thermodesulfobacteriota		3.5
			Nitrospirota		3.3
			GAL15		3.1
			RCP2-54	Remnant	3.3
			MBNT15		3.1
			NB1-j		3.0
			Entotheonellaeota		3.0

Regarding bacterial communities, Planctomycetota and Bacillota phyla were more abundant in the grassland reference state in comparison to the other landcovers, and Chloroflexota, Dependentiae, FCPU426 phyla distinguished the community of the 2-year-old plantation from the others (Table 2, Fig. 2B). Elusimicrobiota and Candidatus Eremiobacterota were significantly more abundant (Kruskal–Wallis; *α* ≤ 0.01) in the 10-year-old plantation as indicated by the LefSe analysis (0.013 ± 0.007% of Elusimicrobiota and 0.002 ± 0.001% of Candidatus Eremiobacterota). In the 24-year-old plantation, distinguishing bacterial phyla included Pseudomonadota, Thermodesulfobacteriota, Nitrospirota, and GAL15. In the remnant forest, RCP2-54, MBNT15, NB1-j, and Entotheonellaeota were the bacterial phyla markedly more abundant. Additionally, the genus *Rhizobium,* belonging to the Pseudomonadota phylum (Willems, 2006), was predominantly detected in the remnant forest soil (Table S2).

Alpha diversity of fungal and bacterial communities within each landcover type was similar. Bacterial communities displayed comparable observed ASVs richness and Shannon-Weaver index (*p* > 0.05) in the grassland and 2, 10, and 24-year-old plantations. In the remnant forest, the observed ASVs richness of bacteria reached a peak of 3,073.5 ± 484.5 and the lowest value of 1,604.3 ± 248.2 in two different transects, making these two sampling locations, both located on the north side of the hill, statistically different from each other. Nevertheless, the Shannon–Weaver index was similar between these samples, with 7.3 ± 0.2 and 6.5 ± 0.2 values, respectively. Fungal communities displayed similar ASVs richness and Shannon-Weaver index within each landcover type.

### Beta diversity of fungal and bacterial communities

**Figure 3:**
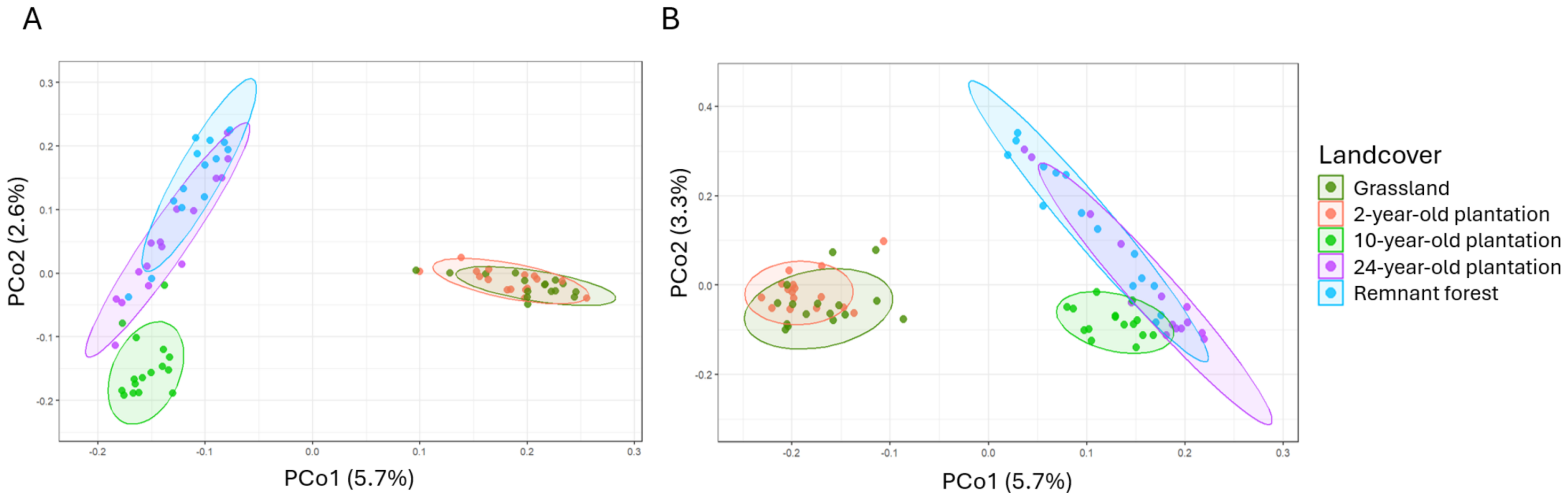
PCoA of the studied microbes. PCoA ordination based on Jaccard dissimilarity index for fungal (A) and bacterial (B) communities belonging to the different landcover types. The two principal components (PCo) holding the highest variance are displayed. From the third PCo (not shown), the variance explained by the axes is below 2% for fungi and 2.5% for bacteria.

The composition of fungal and bacterial communities differed between landcover types. In older *A. mangium* plantations, these communities resembled those found in remnant forests, whereas young plantations had a microbial composition similar to the grassland (Figs. 3A–3B; Table S3). The microbial community of the 24-year-old plantation overlapped with that found in the remnant forest when analysed through PCoA, despite being significantly different from each other as evaluated from the PERMANOVA results (*p* = 0.005 for fungi and *p* = 0.007 for bacteria; Table S3). The 10-year-old plantation showed intermediate composition between grassland and forest sites for both fungi and bacteria (Figs. 3A–3B).

Few fungal and bacterial communities held different variance values (*i.e.,* dispersion) for the beta diversity of each landcover type, requiring a careful evaluation of community composition differences between plantations of distinct ages and reference states. For fungi, only the grassland communities displayed similar beta-diversity dispersion with that of the 2-year-old plantation (PERMDISP; *p* = 0.2), and the remnant forest with the 24-year-old plantation (PERMDISP; *p* = 1) (Table S4). Conversely, all bacterial communities displayed similar variance except for the remnant forest (PERMDISP; Table S4).

#### Fungal and bacterial taxa shared among landcover types

Late stages of forest recovery had more fungal and bacterial ASVs in common with the remnant forest than with the younger plantations and grassland. A total of 18 fungal (0.15% of the total ASVs detected) and 620 bacterial (0.73%) ASVs were shared among all five landcover types. Grassland and 2-year-old plantation had the highest number of common ASVs (550 for fungal and 1,879 for bacterial ASVs). The 24-year-old plantation shared 342 fungal and 1,330 bacterial ASVs with the remnant forest, and 245 fungal and 610 bacterial ASVs with the 10-year-old plantation (Tables S5 and S6).

#### Influences of environmental parameters on soil microbial communities

**Figure 4:**
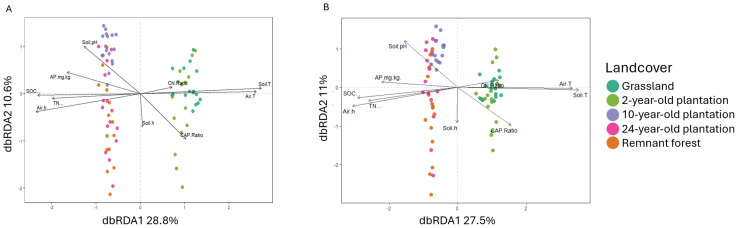
Db-RDA of the studied microbes. Distance-based Redundancy Analysis (db-RDA) ordination of fungal (A) and bacterial (B) communities. SOC, soil organic carbon (%); TN, total nitrogen (%); AP, available phosphorus (mg/kg); C:N, carbon to nitrogen ratio; C:AP, carbon to phosphorus ratio; Air T, air temperature (° C); Air h, air humidity (%); Soil T, soil temperature (° C); Soil h, soil humidity (%).

The composition of fungal and bacterial communities was influenced by soil nutrients and humidity. Fungal communities of early forest recovery stages (grassland and 2-year-old plantation) were separated from the later ones mostly by SOC, air and soil temperature, and TN. Soil humidity, C:AP, and soil pH also contributed to their separation into distinct clusters, as shown by db-RDA (Fig. 4A). For bacteria, soil temperature, air humidity, SOC, AP, and TN explained most of the separation between communities from the grassland, 2-year-old plantation and those from later forest recovery stages and remnant forest (Fig. 4B). Bacterial communities disposition in the multidimensional space was also affected by soil pH, soil C:AP, and air humidity. Specifically, the fungal community was correlated with SOC in the 2-year-old plantation (r_*s*_ = 0.4, *p* = 0.001). Fungi were also correlated with C:AP in the 2- and 24-year-old plantations (r_*s*_ = 0.4, *p* = 0.02 for the 2-year-old plantation and r_*s*_ = 0.4, *p* = 0.01 for the 24-year-old site) (Table S7). In the remnant forests, the composition of fungal communities was influenced significantly by air temperature and humidity (r_*s*_ = 0.3, *p* = 0.01 and r_*s*_ = 0.6, *p* = 0.001, respectively). Soil organic carbon was correlated with the bacterial communities in the 2-year-old plantation (r_*s*_ = 0.4, *p* = 0.001) and grassland (r_*s*_ = 0.3, *p* = 0.03), where they were also associated with AP (mg/kg) (r_*s*_ = 0.4, *p* = 0.04) (Table S8). In the 2-year-old plantation, C:N and C:AP had a significant relationship with the bacterial communities (r_*s*_ = 0.3 for both, *p* = 0.004 and *p* = 0.01, respectively). The C:AP was also correlated with bacterial communities in the 24-year-old plantation (r_*s*_ = 0.4, *p* = 0.02). Finally, bacterial communities in the remnant forest were related to TN (r_*s*_ = 0.2, *p* = 0.04), air temperature and humidity (r_*s*_ = 0.3, *p* = 0.004 and r_*s*_ = 0.5, *p* = 0.003, respectively).

**Figure 5:**
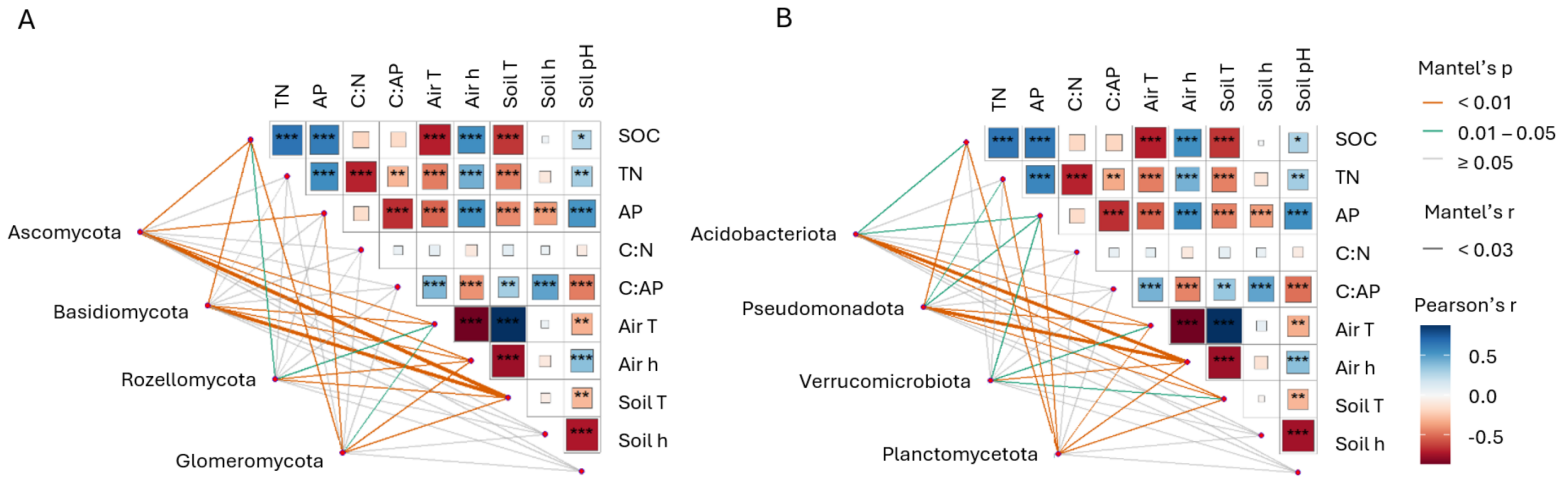
Microbes and environmental parameters. Correlation between the most abundant fungal (A) and bacterial (B) phyla and environmental parameters from the partial Mantel test. SOC, soil organic carbon (%); TN, total nitrogen (%); AP, available phosphorus (mg/kg); C:N, carbon to nitrogen ratio; C:AP, carbon to phosphorus ratio; Air T, air temperature (° C); Air h, air humidity (%); Soil T, soil temperature (° C); Soil h, soil humidity (%). Mantel’s p, *p*-value from the Mantel test. Mantel’s r and Pearson’s r, correlation coefficients.

Several abiotic parameters yielded significant (*p* < 0.01) and high (Pearson’s) correlation coefficients, showing a correlation with the most abundant fungal and bacterial phyla (Figs. 5A–5B). Ascomycota, Basidiomycota, Rozellomycota, and Glomeromycota were predominantly positively correlated with soil temperature (r_*s*_ = 0.3 and *p* = 0.002, r_*s*_ = 0.3 and *p* = 0.002, r_*s*_ = 0.2 and *p* = 0.005, r_*s*_ = 0.1 and *p* = 0.006, respectively). Acidobacteriota, Pseudomonadota, Verrucomicrobiota, and Planctomycetota were positively correlated with air humidity (r_*s*_ = 0.3 and *p* = 0.003, r_*s*_ = 0.3 and *p* = 0.003, r_*s*_ = 0.3 and *p* = 0.001, r_*s*_ = 0.3 and *p* = 0.002, respectively).

### Variation of fungal and bacterial functional groups over forest recovery

**Figure 6:**
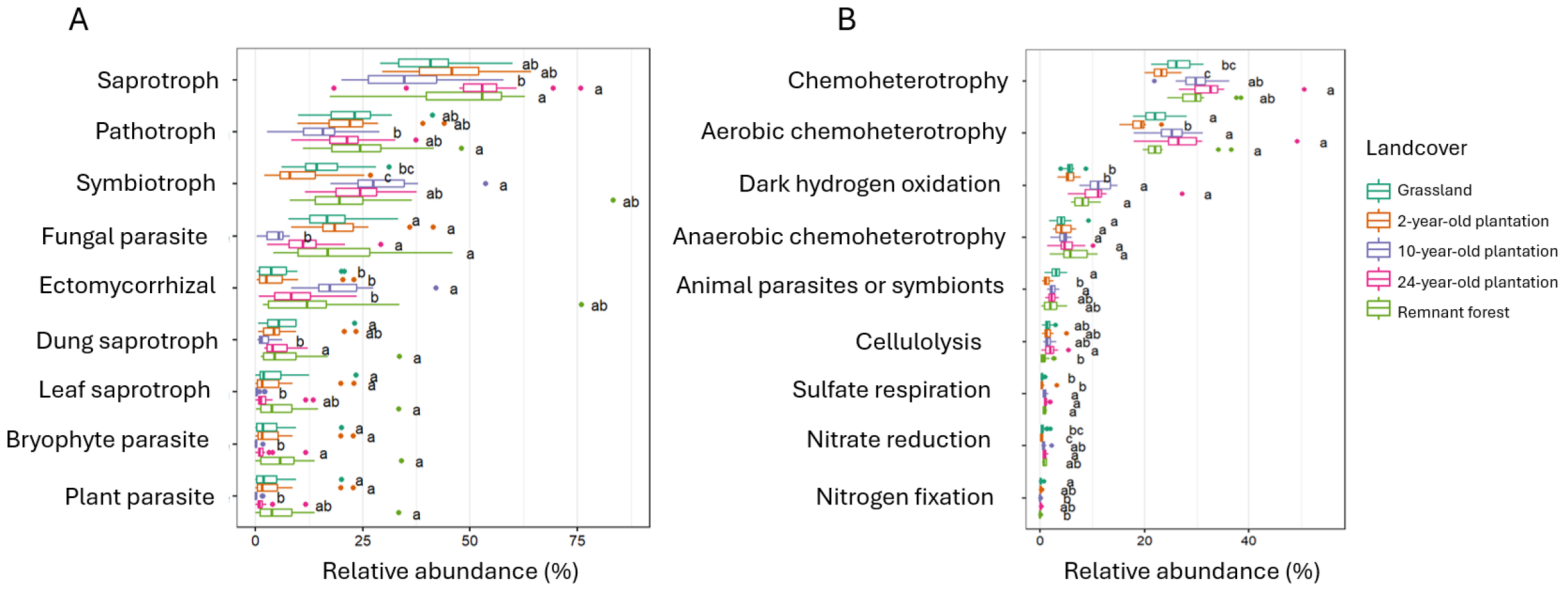
Abundance of microbial functional groups. Relative abundance of fungi trophic modes and guilds (A) and bacterial functional groups (B) in different landcover types. Letters report the statistically different abundance of each group in different landcover types according to the Kruskal-Wallis and Dunn test results among landcover types.

Functional assignment of fungal and bacterial ASVs detected three trophic modes (saprotrophs, symbiotrophs, and pathotrophs), with 23 guilds for fungi and 68 functional groups for bacteria (Table 3). A total of 5,365 ASVs were assigned to a function for fungi (44.7% of the total ASVs observed) and 22,786 (26.9%) for bacteria. The most abundant fungal guilds were parasitic, ectomycorrhizal, and saprotrophic (Table S9, Fig. 6A). For bacteria, chemoheterotrophs, specifically aerobic ones, were the most prevalent bacterial functional group (Table S10, Fig. 6B).

**Table 3:** List of fungal and bacterial functional groups ordered alphabetically and identified through ASVs matches in FUNGuild and FAPROTAX, respectively.

**Fungi guilds**	**Bacteria functions**		
Animal endosymbiont	Aerobic chemoheterotrophy	Aromatic hydrocarbon degradation	Aerobic ammonia oxidation
Animal pathogen	Anaerobic chemoheterotrophy	Chitinolysis	Aerobic anoxygenic phototrophy
Arbuscular mycorrhizal	Animal parasites or symbionts	Dark oxidation of sulphur compounds	Aerobic nitrite oxidation
Bryophyte parasite	Aromatic compound degradation	Dark thiosulfate oxidation	Aliphatic non-methane hydrocarbon degradation
Clavicipitaceous endophyte	Cellulolysis	Human gut	Anammox
Dung saprotroph	Chemoheterotrophy	Human pathogens nosocomia	Anoxygenic photoautotrophy
Ectomycorrhizal	Chloroplasts	Human pathogens pneumonia	Anoxygenic photoautotrophy S oxidising
Endomycorrhizal	Dark hydrogen_oxidation	Hydrocarbon degradation	Chlorate reducers
Endophyte	Fermentation	Invertebrate parasites	Dark sulfide oxidation
Epiphyte	Human associated	Mammal gut	Denitrification
Ericoid mycorrhizal	Human pathogens all	Manganese oxidation	Fumarate respiration
Fungal parasite	Human pathogens meningitis	Methanotrophy	Human pathogens diarrhoea
Leaf saprotroph	Intracellular parasites	Methylotrophy	Human pathogens gastroenteritis
Lichen parasite	Iron respiration	Nitrate ammonification	Human pathogens septicaemia
Lichenized	Nitrate reduction	Nitrate respiration	Ligninolysis
Litter saprotroph	Nitrogen fixation	Nitrite ammonification	Manganese respiration
Orchid mycorrhizal	Oxygenic photoautotrophy	Nitrite respiration	Methanol oxidation
Plant parasite	Photoautotrophy	Nitrogen respiration	Nitrate denitrification
Plant pathogen	Photosynthetic cyanobacteria	Oil bioremediation	Nitrification
Plant saprotroph	Phototrophy	Photoheterotrophy	Nitrite denitrification
Root associated biotroph	Respiration of sulphur compounds	Plastic degradation	Reductive acetogenesis
Soil saprotroph	Sulfate respiration	Predatory or exoparasitic	Sulphur respiration
Wood saprotroph	Ureolysis	Xylanolysis	

The functional profile of fungal and bacterial communities became increasingly similar to the remnant forest during natural regeneration (Figs. S7A–S7B). Saprotrophic fungi were the prevalent trophic mode across all landcover types, with higher abundance in the 2, 24-year-old plantation and remnant forest (46 ± 9%, 52 ± 13%, and 48 ± 14%, respectively) compared to grassland (41 ± 9%) and 10-year-old plantation (35 ± 10%) (Tables S9 and S11). Pathotrophic fungi were similarly abundant in all sites except in the 10-year-old plantation, where their relative abundance was lower (*p* < 0.05). The third most abundant feeding strategy for fungi was the symbiotrophic, which was equally high in the 10, 24-year-old plantations and remnant forest (average within these landcover types of 25.8 ± 12.4%). Such a type of nutrition was lower in the grassland and 2-year-old plantation (average within these landcover types of 13.7 ± 7.4%; *p* < 0.05) compared to the older sites. Overall, the 24-year-old plantation presented the highest number of fungal functional groups (23).

For bacteria, chemoheterotrophy was significantly more abundant in the 24-year-old plantation compared to all the other landcover types (*p* < 0.05) (Tables S10–S12). Within this group, the aerobic chemoheterotrophy similarly occurred in the grassland and remnant forest, but was dominant in the 24-year-old plantation (*p* < 0.05). Anaerobic chemoheterotrophy was consistently present among the phases of forest recovery. Dark hydrogen oxidation was more abundant in the 10- and 24-year-old plantations (11 ± 2% and 11 ± 5% respectively; *p* < 0.05) and remnant forest (8 ± 2%) than in the 2-year-old plantation and grassland (both 6 ± 1%; *p* < 0.05). Sulfate respiration was more common in the latest stages of recovery than in early periods (*p* < 0.05), and nitrate reduction displayed a similar pattern. Nitrogen-fixing bacteria were more present in the grassland compared to the 10-year-old plantation and remnant forest (*p* < 0.05). Overall, the remnant forest had the highest number of represented bacterial functional groups (61; *p* < 0.05).

Visualization of the similarities between functional profiles of microbial communities across landcover types highlighted three outliers (two related to fungal communities and one to those of bacteria, respectively). These outliers corresponded to samples from the 10-year-old plantation, and remnant forest for fungi, and from the 24-year-old plantation for bacteria. Analysis of the relative abundances of functional groups within these samples compared to the other samples belonging to the same landcover type showed a marked difference in fungal symbiotrophs, particularly ectomycorrhizal fungi (Table S13), and in bacterial functions such as dark hydrogen oxidation and chemoheterotrophy functions (Table S14), which were already the most represented across the landcovers (Table S12).

Fungal and bacterial functional groups exhibited distinct correlations with environmental parameters across different landcover types. Ectomycorrhizal fungi were correlated with soil pH in the 24-year-old plantation (r_*s*_ = 0.54, *p* = 0.03) (Fig. S8A). In the 2-year-old plantation, endomycorrhizal fungi were negatively associated with soil pH (r_*s*_ = −0.47, *p* = 0.06) and soil humidity (r_*s*_ = −0.49, *p* = 0.05), but in the grassland, they were positively correlated to air temperature (r_*s*_ = 0.50, *p* = 0.05) and C:AP (r_*s*_ = 0.64, *p* = 0.007), and negatively associated with AP (r_*s*_ = −0.61, *p* = 0.01). The anaerobic chemoheterotrophy bacterial function was inversely correlated with AP in grassland (r_*s*_ = −0.6, *p* = 0.02), while aerobic chemoheterotrophy was positively and strongly correlated with C:N in the remnant forest (r_*s*_ = 0.7 and *p* < 0.01), and negatively with soil temperature in the same landcover type (r_*s*_ = −0.53 and *p* = 0.04) (Fig. S8B). In the remnant forest, sulphate respiration (r_*s*_ = −0.52, *p* = 0.05) and cellulolysis (r_*s*_ = −0.55, *p* = 0.03) were negatively associated with SOC. In the same landcover type, dark oxygen oxidation was negatively correlated with TN (r_*s*_ = −0.55, *p* = 0.03), while fermentation was positively correlated with both SOC (r_*s*_ = 0.64, *p* = 0.009) and TN (r_*s*_ = 0.55, *p* = 0.03). Taxa capable of becoming parasites of invertebrates were present in all landcover types, but only significantly correlated with SOC (r_*s*_ = −0.54, *p* = 0.03), TN (r_*s*_ = −0.63, *p* = 0.01), C:N (r_*s*_ = 0.65, *p* = 0.008), and AP (r_*s*_ = −0.62, *p* = 0.01) in the 10-year-old plantation.

## Discussion

### High and distinct taxonomic diversity of fungi and bacteria over forest recovery trajectory

Our study shows that grasslands and remnant forests have similar ASVs richness but differ in their microbial community composition. Observed dissimilarities were largely driven by heterogeneous dispersion rather than true structural differences. The value and similarity of the taxonomic diversity we found over successional changes agree with past research on dry and moist tropical forests recovery (Saltonstall et al., 2025). Furthermore, our results display a comparable microbial richness and Shannon-Weaver index across natural regeneration. This evidence contradicts the assumption that *I. cylindrica* “degraded” grasslands, which have lower plant diversity compared to remnant forest (Vivian et al., 2026), hold and impose low microbial biodiversity values (Holm et al., 1977; MacDonald, 2004; Brewer, 2008; Daneshgar & Jose, 2009). Thus, we highlight the potential of *I. cylindrica* grasslands to support numerous ecosystem services, as previously observed (Zhao, Liu & Wu, 2020).

**Figure 7:**
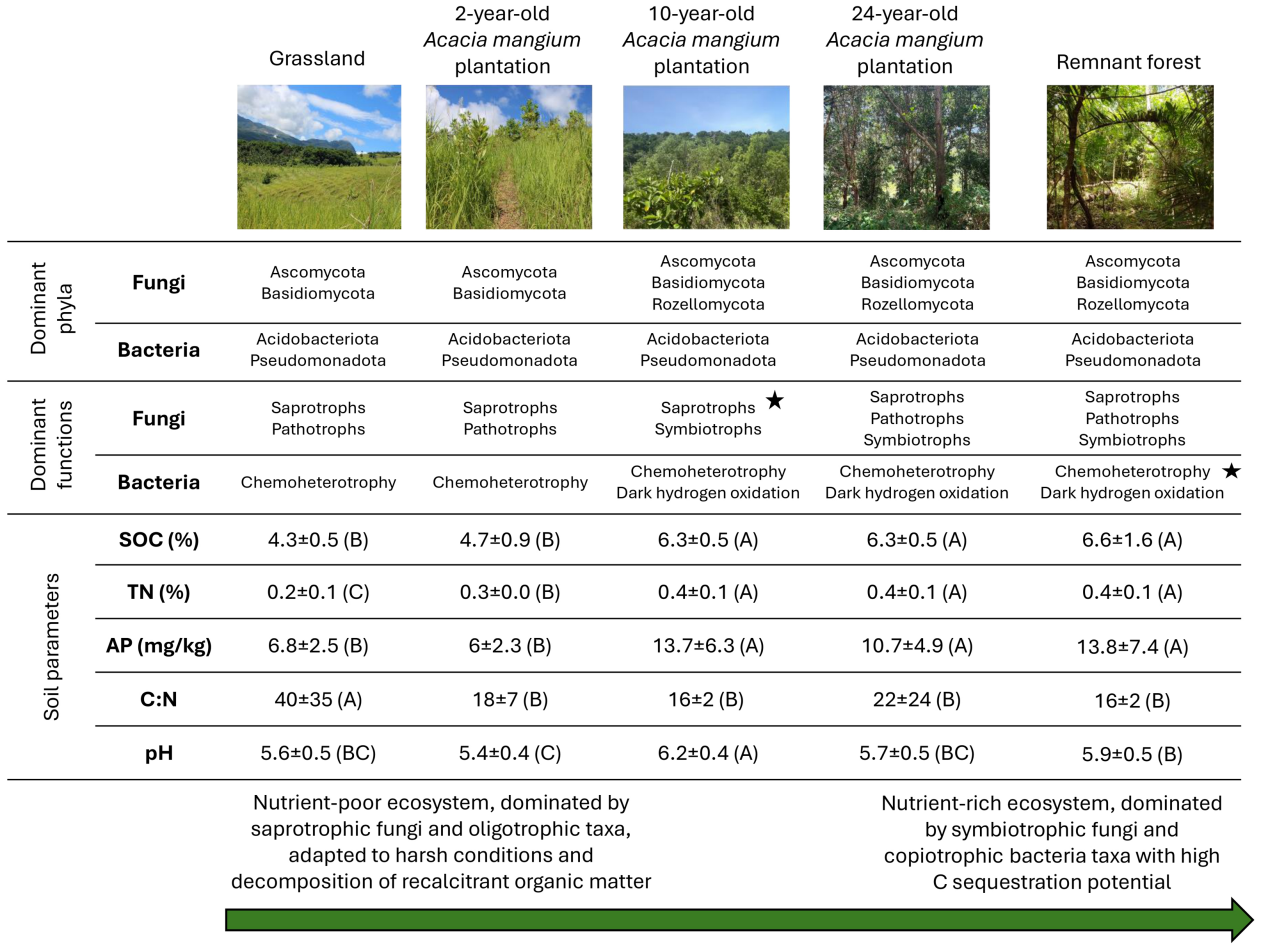
Summary of microbes and environmental variations. Overview of dominant fungal and bacterial phyla (*i.e.,* with the highest relative abundance) and their inferred functions across the natural regeneration in *A. mangium* plantations of 2, 10, and 24 years, and reference states of grassland and remnant forest. Stars highlight the landcover displaying the highest number of functions. Capital letters in brackets express the significant difference (*p* < 0.05) between the relative soil parameters in each landcover type. SOC, soil organic carbon; TN, total nitrogen; AP, available phosphorus.

We found clear evidence of a transition from relatively nutrient-poor ecosystems to organic matter-rich environments from the 10-year-old plantation onwards, mirrored by changes in the abundance of fungal phyla associated with decomposition. The Ascomycota and Basidiomycota fungal phyla were highly represented in the grassland (Fig. 7), consistent with their ability to break down recalcitrant organic matter (Ma et al., 2013) and the presence of the former in degraded conditions (Gourmelon et al., 2016). Conversely, in advanced restoration stages, the Mortierellomycota phylum was more abundant than in younger naturally regenerating plantations, potentially reflecting its role in mineral phosphorus dissolution (Gai et al., 2021) and vegetation residue degradation (Osono, 2005). Overall, the presence and relative abundance of the described fungal phyla can provide valuable indicators for monitoring ecosystem evolution (Fernandez Nuñez et al., 2021; Manici et al., 2024), enabling the assessment of restoration progress and identifying potential need for interventions.

We observed that bacteria adapted to harsh and nutrient-rich conditions marked the transition from grassland to the *A. mangium* plantation. The Bacillota phylum (formerly Firmicutes; Oren & Garrity, 2021), which is adapted to harsh conditions (Deng et al., 2025), was one of the dominant phyla in the grassland, as assessed in previous studies (*e.g.*, Wang et al., 2019). Naturally regenerating plantations were characterised by a common presence of the Elusimicrobiota, which was relatively abundant in older plantations, consistent with a nutrient-rich soil (Liu et al., 2024). The FCPU426 bacterial phylum, capable of cellulose decomposition (Doud et al., 2020), was also common in the older plantations. Finally, the MBNT15 phylum was present in the remnant forest, potentially contributing to the complete mineralisation of organic substances (corroborated by its correlation with SOC; Fig. S9) (Begmatov et al., 2022). Thus, bacterial phyla presence or relative abundance may serve as indicators of ecosystem progression toward a nutrient-rich forest state.

Despite sharing multiple taxa between stages of recovery and their relative reference state, the composition of soil microbial communities differed significantly over the restoration timeframe. Given that replication and sampling adequately captured differences between landcover types, significant differences in community composition may reflect variance in the data or be driven by soil nutrients and aboveground characteristics (*e.g.*, canopy coverage, tree species diversity). This aligns with past findings showing microbial taxa are dependent on environmental parameters (Dini-Andreote et al., 2015; Yang, Dou & An, 2018; Zhou et al., 2022; Li et al., 2023; Yan et al., 2023). Interpretation is limited, however, by the substantial proportion of “unknown” ASVs, and by the restricted geographical area assessed. Thus, our study may represent the expected results in similar conditions, but further studies would be needed to confirm this. More generalized conclusions warrant further studies in different biomes.

The results of this study suggest that the taxonomic composition of soil fungal and bacterial communities can potentially be used to monitor forest restoration, along with other environmental analyses. Still, a comprehensive understanding of the microbial community is constrained by the PCR biases, different sequencing parameters utilised (Polz & Cavanaugh, 1998; Aird et al., 2011), and available genetic information to identify the sequenced ASVs. Furthermore, the heterogeneous distribution of soil nutrients may hinder the detection of microbial taxa and understanding of their community variation (Simon et al., 2024). Nevertheless, fungal and bacterial taxa can still indicate forest recovery (*e.g.*, Fernandez Nuñez et al., 2021; Zhang, Li & Ji, 2023), with our results corroborating such evidence. Importantly, while communities differed in composition from each other, they demonstrated a clear separation between early and late phases of forest recovery, as visible in PCoA and db-RDA ordination (Figs. 3A–3B; Figs. 4A–4B). For fungi, observed patterns may be influenced by community heterogeneity; for bacteria, environmental factors likely contributed to significant variation. The disposition in multidimensional spaces highlights the possible role of indicators not only of specific taxa, but of the whole community composition, for the identification of the restoration trajectory. In the studied natural regeneration of the plantations, soil fungal and bacterial communities increasingly resembled those of the remnant forest, as expected in our hypothesis. With respect to previous studies focused on *A. mangium* plantations (*e.g.*, Wei et al., 2024), our analyses expand knowledge on the variation of microbial communities over time in a context of exotic plantations in the tropics. Furthermore, given the insights on the restoration trajectory expressed by the variation of microbial communities, as recent studies displayed (*e.g.*, Gellie et al., 2017; Adamo et al., 2021), we suggest the adoption of this type of assessment for forest monitoring.

### Above- and belowground ecosystem parameters influence fungal and bacterial communities

Similar soil nutrients, tree communities, and aboveground cover may explain the large number of ASVs shared by the early stages of natural regeneration and grassland, and the later phases of recovery and remnant forest, respectively. The marked difference in the environmental characteristics over the natural regeneration, transitioning from an open to a closed and shaded forest ecosystem, is consistent with the discussed separation (between early and late stages of recovery) and similarity (within early and late stages of recovery, respectively) of the microbial communities, both in terms of shared ASVs and community composition.

Previous studies found soil fungi and bacteria to be largely affected by the vegetation composition (*e.g.*, Fernandez Nuñez et al., 2021; Silva et al., 2023), soil nutrient concentration (Koorem et al., 2014), and microclimatic conditions (*e.g.*, Fernandez Nuñez et al., 2021; Graham & Knelman, 2023; Lemke & De Salle, 2023). Our results are consistent with these studies, as indicated by db-RDA ordination and the importance of soil nutrients for the separation of the communities over the horizontal and vertical axes. In our study, bulk topsoil organic carbon, slightly acidic pH values, and TN concentration were particularly associated with the distinction of microbial community change over ageing *A. mangium* plantations. In the last decade, it was found that uneven distribution of soil nutrients (Jackson & Caldwell, 1993), combined with the different chemical composition of plant tissues (Hobbie, 1992; Hobbie et al., 2007; Dinakaran & Krishnayya, 2008; Yang et al., 2023), likely promote microbial diversity, as supported by both our data and previous studies (*e.g.*, Adamo et al., 2021; Lu et al., 2023a). Along the restoration timeframe, we found a high variance in microbial community composition, possibly indicating the occurrence of different ecological niches (Jackson & Caldwell, 1993; Hodge, 2006). The distinction between communities is also emphasised by the high bacterial taxonomic variability, as displayed in the 24-year-old plantation and remnant forest (1,757.9 ± 495.0 and 2,184.5 ± 691.6 ASVs, respectively; Table 1). Yet, these two landcover types still differed in microbial composition and diversity, indicating that full recovery requires longer timescales than the 24 years studied here. Fungal community differences likely reflect environmental variation, as heterogeneity between the 24-year-old plantation and remnant forest remained similar, ruling out dispersal effects as the main driver of variance.

Fungal phyla appeared to be more responsive to environmental variation, consistent with past research showing the resilience of bacteria to micro-climatic changes (Uroz et al., 2016; Wang et al., 2019; Mirza et al., 2020). Fungi have delicate physical structures (*i.e.,* hyphae), whereas bacteria can withstand harsh conditions through chemicals (*e.g.*, enzymes) and biofilms (Wang et al., 2019). Thus, when early evidence of ecosystem recovery is needed, fungi may be preferred, given their higher sensitivity to environmental changes compared to bacteria (Yan et al., 2018). Conversely, in long-term monitoring, and when funding restricts the study to one microbial group, the analysis of bacterial communities may be favoured, since more studies have been developed on their characteristics (*e.g.*, taxonomy, metabolism), and their influence on ecosystem functioning and health, compared to fungi. Still, the shared influence of C:AP on fungal and bacterial community composition in the 2 and 24-year-old plantations, and for C:AP and SOC in the 2-year-old plantation, highlights common factors possibly affecting both microbial groups. Therefore, while fungi may provide early evidence of ecosystem modifications, we suggest the consideration of the whole microbial community and fine-scale variations in soil nutrients in ecosystem monitoring to take advantage of their indicator roles.

Bacteria may aid in the recognition of the recovery potential of the studied area by informing on the soil physical disruption and land-use history. The GAL15 bacterial phylum is usually found to be abundant in subsurface soil horizons (Brewer et al., 2019). In our study, its presence in the topsoil of the 24-year-old plantation may indicate disturbances affecting the soil strata organisation (*e.g.*, typhoons uprooting trees) and the need for substrate stabilisation to ensure the recovery of slow-growing native trees. Additionally, the *Rhizobium* genus (belonging to the Pseudomonadota phylum; Willems, 2006) characterised the remnant forest topsoil. Thus, we hypothesize the association of this taxon with native tree species besides the exotic *A. mangium* (National Research Council, 1983), such as *Sindora supa*, or *Pterocarpus indicus* (Thomson, 2006), but also the influence of the land-use history (Bonner et al., 2019), assuming past cultivation or occurrence of these tree species.

### Recovery of fungal and bacterial functional traits during reforestation using *Acacia mangium*

Fungal and bacterial communities developed symbiotroph and copiotrophic functional traits, respectively, during the studied forest recovery. While acknowledging the low proportion of fungal and bacterial taxa for which their putative functional role was successfully assessed, a pattern of fungal and bacterial function transition was evident. This dynamic reflects tree community recovery and the association with ecto- and endomycorrhizal fungi of *A. mangium* (Aggangan, Moon & Han, 2010), and native species (Smits, 1994; Lee, 2006; Tedersoo et al., 2006; Aggangan, Moon & Han, 2010). In our study, fungal communities recovered the symbiotroph traits within the first 10 years after plantation establishment. The abundant variation of such groups from early to late stages of forest regeneration aligns with previous findings in revegetation and afforestation contexts (*e.g.*, Fernandez Nuñez et al., 2021; Zhang, Li & Ji, 2023). Similar to fungal symbiotrophic taxa, the increase of copiotrophic bacterial taxa over forest recovery is well known (*e.g.*, Liang et al., 2021; Fernandez Nuñez et al., 2021; Liao et al., 2023; Vivian et al., 2025) and found in our research.

Changes in nutrient cycling over the natural regeneration are potentially indicated by the strong correlations of ecto- and endomycorrhizal fungi with environmental parameters. While not being the dominant guild, endomycorrhizal fungi must be considered, since their relative abundances may not equal the importance of their role in ecosystem dynamics (Jones & Lennon, 2010; Campbell et al., 2011; Shade et al., 2012; Shade et al., 2014). Specifically, endomycorrhizal fungi are particularly important for phosphorus (P) uptake (Begum et al., 2019; Lu et al., 2023b), making them crucial in the early phases of plantation establishment and the onset of natural regeneration. This is supported by the positive correlation with available phosphorus (AP) in the 2- and 10-year-old plantations, with negative correlations in other landcover types. Conversely, ectomycorrhizal fungi have been found to enhance the acquisition of numerous nutrients by plants (Courty et al., 2010) and influence their water uptake (Lehto & Zwiazek, 2011; Sevanto et al., 2023). According to our results, we hypothesise that in the grassland and 2-year-old plantation, where SOC is low, ectomycorrhizal relative abundance may increase quickly, providing numerous resources to their associated vegetation. Then, with the presence of a more stable and nutrient-rich state (*i.e., A. mangium* 10-, 24-year-old plantation and the remnant forest), endomycorrhizal fungi may outcompete ectomycorrhizal fungi. This shift would be due to higher demand for P, thus the preference of tree species for endomycorrhizal fungi, specialised in such a soil nutrient acquisition (increasingly limiting the growth of vegetation over the forest succession; Xue et al., 2022), unlike the generalist ectomycorrhiza. Finally, during *A. mangium* natural decay (around the 20th–30th year; Guzman, Lawrence & Marler, 1997; Krisnawati, Kallio & Kanninen, 2011), the tree community turnover may impose a return to the conditions favouring the association with ectomycorrhiza, given their capability to increase the availability of numerous nutrients and stabilise soil.

Bacterial functions related to soil fertility and nutrient cycles seemed to be restored over natural regeneration. Sulfate respiration recovered from the 10th year of the natural regeneration in *A. mangium* plantations, possibly leading to improved nutrient cycles (Demin et al., 2024) and the degradation of organic carbon (Wartell, Boufadel & Rodriguez-Freire, 2021). Nitrate reduction, a fundamental process for balancing nitrogen concentrations in the ecosystem (Kamp et al., 2015), also recovered with activity inversely related to C:N ratio (Bengtsson, Bengtson & Månsson, 2003). Furthermore, bacterial denitrification recovered to levels displayed in the remnant forest by 24 years from the plantation establishment. Initially, the concentration of nitrogen (N) is one of the most important limiting factors for tree growth (Jiang et al., 2018; Xue et al., 2022; Song et al., 2023). At this stage, bacterial communities are involved in N fixation and other nutrient acquisition, aiding plant growth. Then, the factors limiting tree growth become P (Xue et al., 2022), light (Fransson, Brännström & Franklin, 2021), and space competition. In this phase, bacterial denitrification balances the atmospheric level of the N element previously sequestered (Gundersen, 1991) while buffering pH variations (Robertson, 1989). Still related to the N-cycle, nitrite-oxidising bacteria (Nitrospirota; Mehrani et al., 2020) were also highly abundant in the nutrient-rich conditions of the 24-year-old plantation, likely affecting the N-cycle and related consequences on nutrient availability. Thus, the development of specific bacterial processes may promote tree species coexistence (Huston & DeAngelis, 1994; Barot, 2004), allowing for the development of the rich tree community observed in the 24-year-old plantation and remnant forest (see Vivian et al., 2026).

Our results show that functional diversity seemed to recover faster for fungi compared to bacteria. Fungal functional richness was at its highest in the 24-year-old plantation, highlighting the “functional bridge” role of such a stage of forest natural regeneration for the return to historical functional conditions of the fungal community. This microbial group shifted from being dominated by saprotrophic to symbiotrophic fungi. Conversely, the highest bacterial functional richness was displayed in the remnant forest, consistent with their slow response to environmental changes (Uroz et al., 2016; Wang et al., 2019) and a possible enrichment of their functional traits in a more stable ecosystem. For instance, sulphur and manganese respiration functions were only present in the remnant forest. These processes can rely on numerous substrates as electron acceptors compared to functions present in other landcover types (Nealson & Saffarini, 1994; Muyzer & Stams, 2008). This flexibility underlies the higher niche filling of bacterial communities in the remnant forest compared to earlier phases of forest recovery, assuming the creation of ecological niches over the reforestation dynamic (Lu et al., 2023a). However, the large proportion of ASVs with unassigned functions may have limited detection of similar roles in other landcovers, likely represented by taxa yet to be sequenced. We therefore emphasise the need to study previously unknown microbial taxa to identify key organisms supporting ecosystem functioning.

### C sequestration and forest restoration assessment from soil fungal and bacterial community analysis

Restoring fungal and bacterial communities is fundamental to enhancing carbon sequestration and mitigating climate change. Especially important for this purpose are ectomycorrhizal fungi (mainly Basidiomycota and Ascomycota; Tedersoo, May & Smith, 2010), which we found particularly abundant in the 24-year-old plantation. These organisms are known to protect the roots of plants and enhance nutrient and water uptake (Courty et al., 2010; Lehto & Zwiazek, 2011; Sevanto et al., 2023), while improving C sequestration in the soil through their biomass and stable soil aggregate formation (Zhang et al., 2025). Furthermore, the Mortierellomycota phylum, highly abundant in advanced states of forest restoration, seems to enhance the soil C cycle (Han et al., 2022), playing a key role in the initial stages of organic matter decomposition (Koechli et al., 2019; Clocchiatti et al., 2020) and subsequent nutrient release, which enables tree growth. Therefore, the high abundance of the described fungal phyla in the advanced state of forest restoration in our study may suggest a trajectory toward an ecosystem that favours C storage.

Bacterial functional contribution to C sequestration is represented by multiple taxa and processes. Dark hydrogen-oxidation bacteria (Bacillota, Actinobacteriota, Proteobacteria phyla; Jordaan et al., 2020; Li et al., 2025) retrieve energy by consuming atmospheric H_2_, with subsequent CO_2_ sequestration (Jordaan et al., 2020; Greening, Islam & Bay, 2022). Through this process, some bacteria oxidise methane, replacing it with CO_2_, which has a much lower global warming potential than the former (IPCC, 2021). Dark hydrogen oxidation is positively influenced by soil humidity (Jordaan et al., 2020), and we found that this parameter increases over the natural regeneration timeline (Fig. 7 and Table S1). This putative bacterial function was particularly abundant in the 24-year-old plantation outlier (Fig. S7B and Table S14), at the expense of anaerobic chemoheterotrophy, for which an opposite correlation with environmental parameters compared to the one displayed by dark hydrogen oxidation was found, and through which harmful greenhouse gases can be produced (Jurtshuk Jr, 1996). Thermodesulfobacteriota and Chloroflexota phyla also recovered with plantation age. The former can express sulphate respiration (Vick et al., 2010), potentially mitigating greenhouse gases *via* competition with methanogens (Michas et al., 2020; Scholz et al., 2020). Conversely, the latter is known for fixing C even in harsh environments (van der Meer et al., 2003; Klatt et al., 2013; Kawai et al., 2021), which may still be present as microclimatic conditions over the forest recovery. Moreover, the RCP2-54 phylum was highly abundant in the remnant forest, likely reflecting its adaptation to humid microoxic or anoxic soil conditions (Table S1; Le Mer & Roger, 2001), for which we found evidence in this reference state. This phylum seems to degrade alkanes and is involved in CH_4_ oxidation (Murphy et al., 2021), making it a potentially useful tool for mitigating methane emissions (Gontijo et al., 2024). Still in the remnant forest, we found the Entotheonellaeota phylum, possibly contributing to the cycling of C and micronutrients (De Sousa Lopes et al., 2021; Jia et al., 2023). Given our results, we recommend further studies to disentangle the relationships between environmental parameters and the examined metabolic processes, to understand contributions to the mitigation of greenhouse gas production and mechanisms for removing or avoiding them.

Monitoring forest restoration may enable the identification of the best conditions for the expression of microbial C sequestration. In our study, the C sequestration potential of the abundant C-fixing bacteria from the 10th year after planting may cooperate with the ability of *A. mangium* to rapidly provide shade and nutrients, given its fast-growing traits and symbiotic relationships with soil microorganisms (National Research Council, 1983; Hegde, Palanisamy & Yi, 2013). Overall, our findings show that the natural regeneration in *A. mangium* plantations may enhance C sequestration by promoting the recovery of microbial taxa and associated functions.

## Conclusions

Soil eDNA analysis revealed a trajectory of the recovery of microbial communities and functions in naturally regenerating *A. mangium* plantations, highlighting the potential of these cultivations for tropical forest restoration and climate change mitigation. Although total taxonomic richness remained fairly constant over the forest recovery, the community composition varied from being rich in taxa adapted to harsh conditions to being dominated by microorganisms thriving in nutrient-rich environments. The functions expressed by the identified taxa also indicated mechanisms of forest regeneration through time, with fungi appearing to respond faster than bacteria to ecosystem modifications. Overall, a highly diverse microbial community seemed to be associated with changes in above- and below-ground environmental parameters and may contribute to C sequestration, nutrient cycling stabilisation, and the promotion of tree species coexistence and growth. Given the high variation in microbial community composition and budget constraints of each project, restoration planning and monitoring should strategically focus on either soil fungi or bacteria, given their potential as indicators of ecological processes and successful forest recovery. This study expands knowledge on the microbial community associated with exotic plantations of *A. mangium* using a space-for-time approach, and illustrates how soil eDNA analysis can provide early insights into ecosystem changes. Fungal and bacterial community composition and function can be integrated with vegetation and soil nutrient data to better characterise forest recovery trajectories.

##  Supplemental Information

10.7717/peerj.21048/supp-1 Supplemental Information 1 Supplementary Material

## Additional Information and Declarations

### Field Study Permissions

The following information was supplied relating to field study approvals (*i.e.*, approving body and any reference numbers): Field experiments were approved under the Project Tarsier agreement with local Peoples Organisations in Biliran, following Free Prior and Informed Consent principles. Furthermore, the fieldwork was approved by the University of the Sunshine Coast.

### DNA Deposition

The following information was supplied regarding the deposition of DNA sequences: The raw sequences are available at NCBI Sequence Read Archive: PRJNA1295595.

### Data Availability

The following information was supplied regarding data availability: The raw sequences are available at NCBI Sequence Read Archive: PRJNA1295595. The code is available at Zenodo and GitHub: - Jenny Vivian. (2025). JennyVivian99/Soil_microbes_AcaciaMangium_natural_ regeneration: Recovery of Soil Microbiota in Naturally Regenerating Acacia mangium Ecosystems (Soil_AcaciaMangium_plantations_microbes). Zenodo. https://doi.org/10.5281/zenodo.17393365. - https://github.com/JennyVivian99/Soil_microbes_AcaciaMangium_natural_regeneration
