# Supplementary material for: Recovery of soil microbiota in naturally regenerating Acacia mangium ecosystems
Source: PeerJ. 2026 Apr 13;14:e21048. doi: 10.7717/peerj.21048 (PMC13086022; doi:10.7717/peerj.21048)
Supplement: Supplemental Information 1 [file peerj-14-21048-s001.docx]

**Supplementary material**

**Figures**


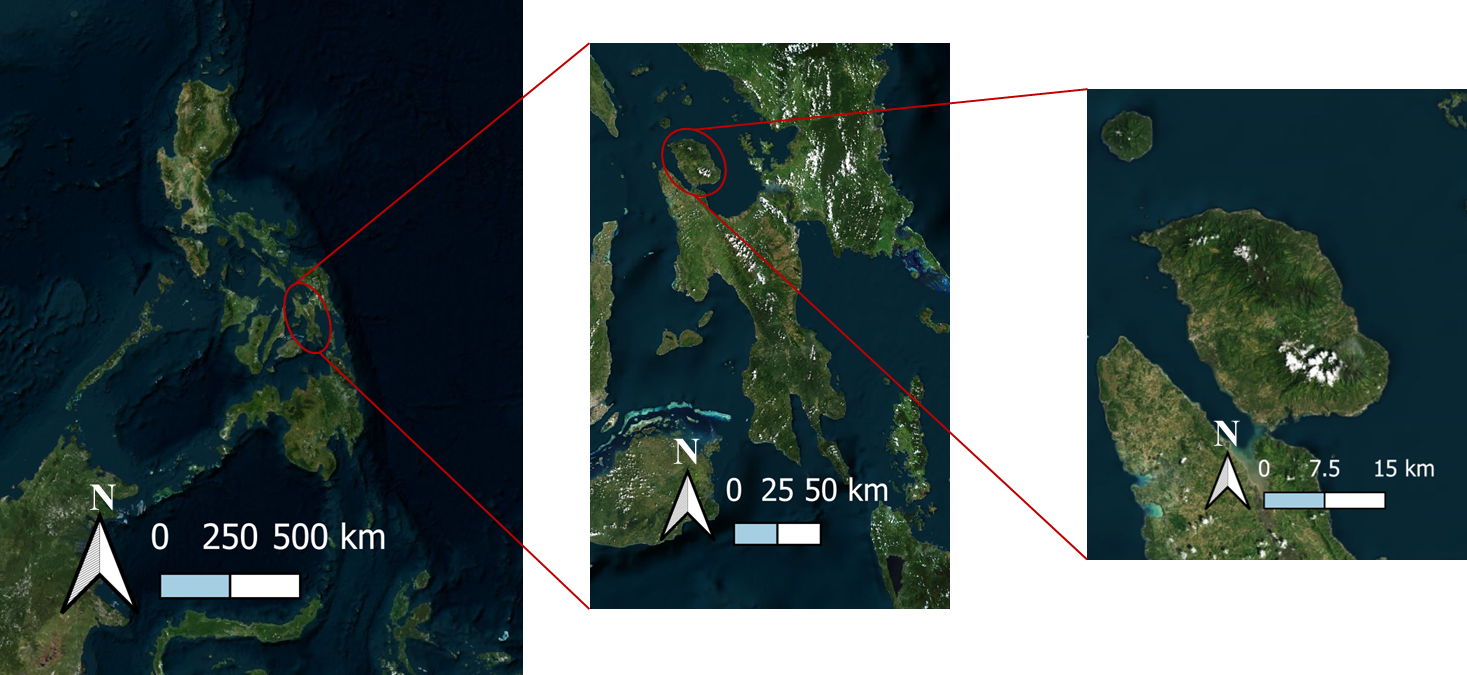


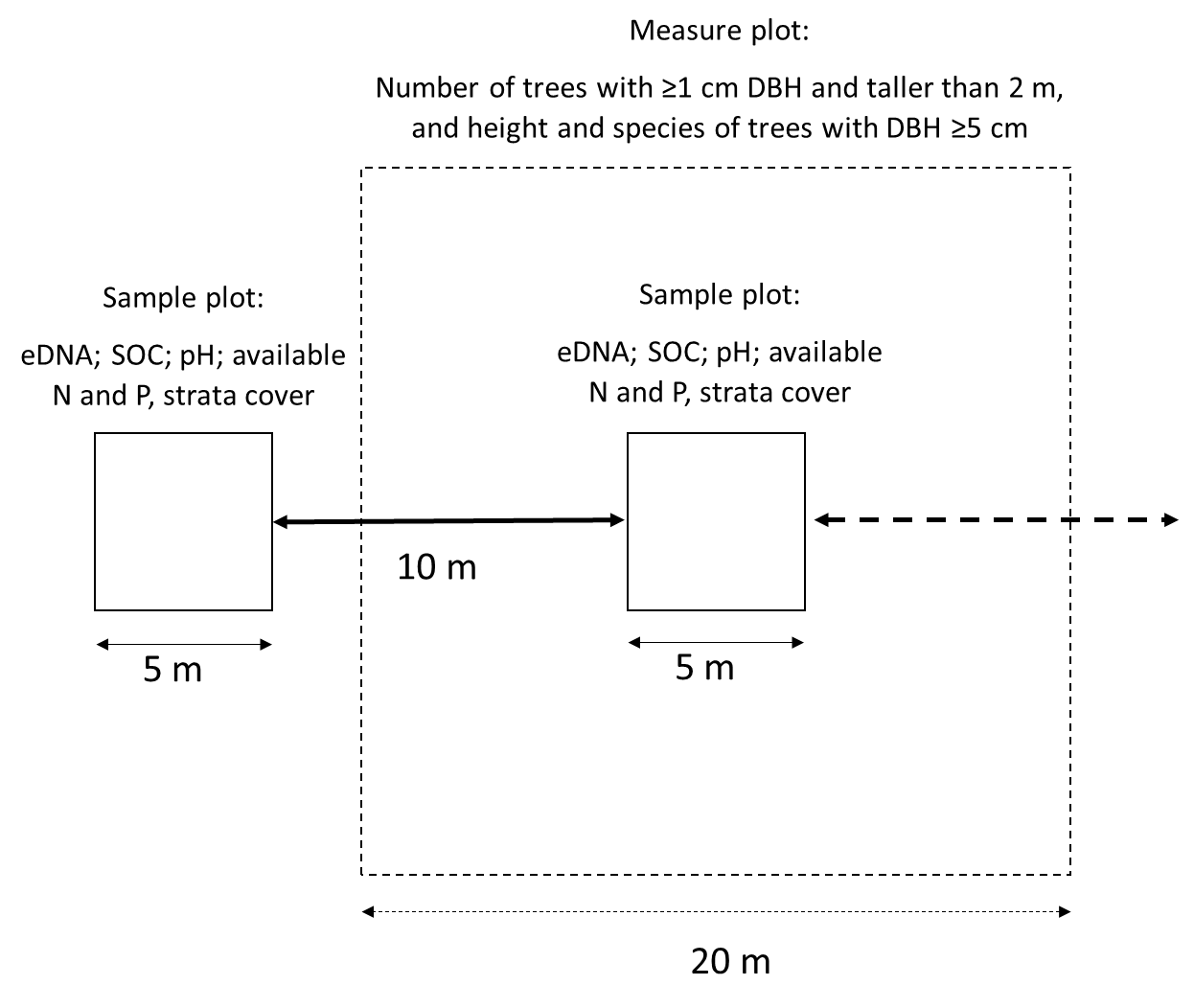
**Fig. S1** Map of the area of the study. From left to right: Philippines, Eastern Visayas, Biliran (island where the study plots are located). Map created using QGIS and Microsoft Bing Maps Satellite Imagery.

**Fig. S2** Plot dimensions, parameters evaluated and distances between plots.


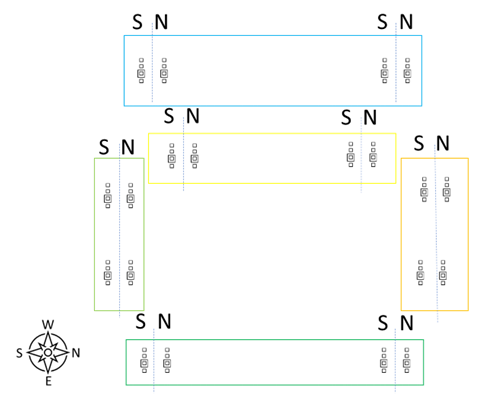


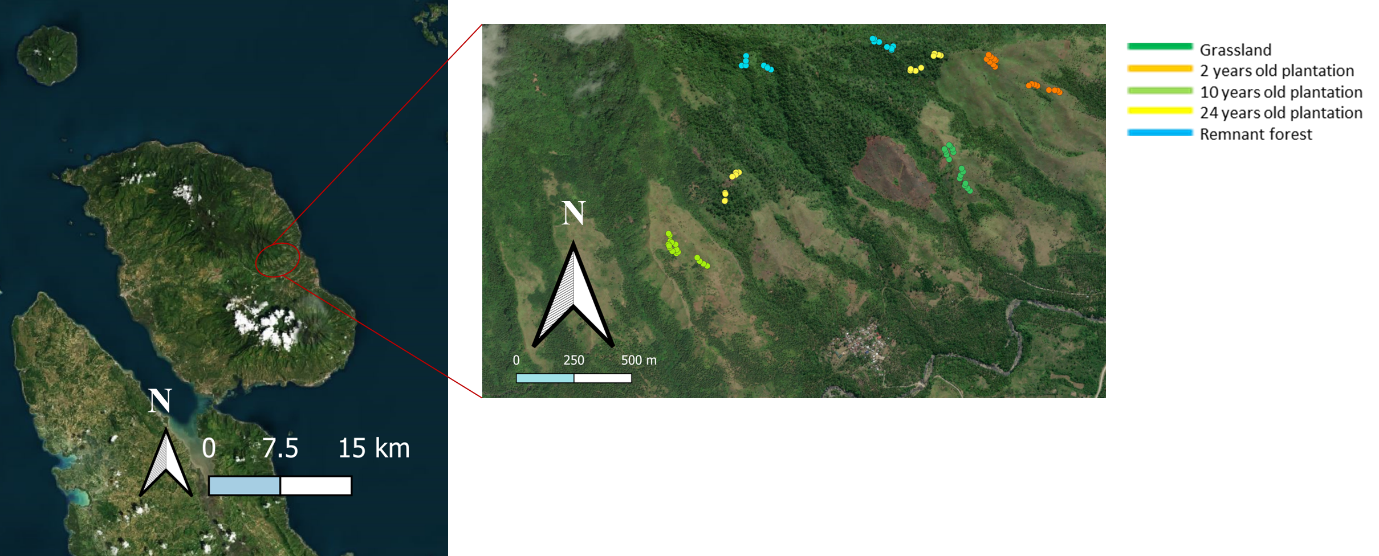
**Fig. S3** Plot disposition. Single squares indicate plots where eDNA and soil data were collected (i.e., “Sample plot” in Fig. S3). Double squares indicate plots where data on the tree community were also assessed (i.e., “Measure plot” in Fig. S3). For tree community data, see Vivian et al. (2026). Southern- and Northern-facing transects are indicated as “S” and “N”, respectively. Colours represent the different landcover types (dark green: grassland; orange: 2-year-old plantation; light green: 10-year-old plantation; yellow: 24-year-old plantation; light blue = remnant forest).

**Fig. S4** Plots in the study area. Map created using QGIS and Microsoft Bing Maps Satellite Imagery.

**
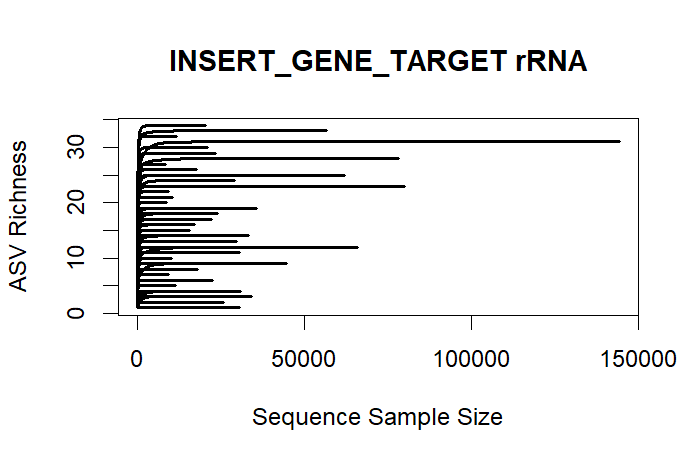
**

**Fig. S5** Rarefaction curve of fungi.


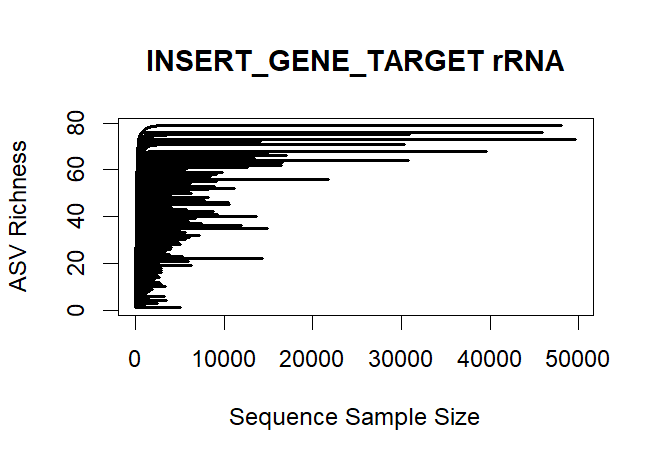


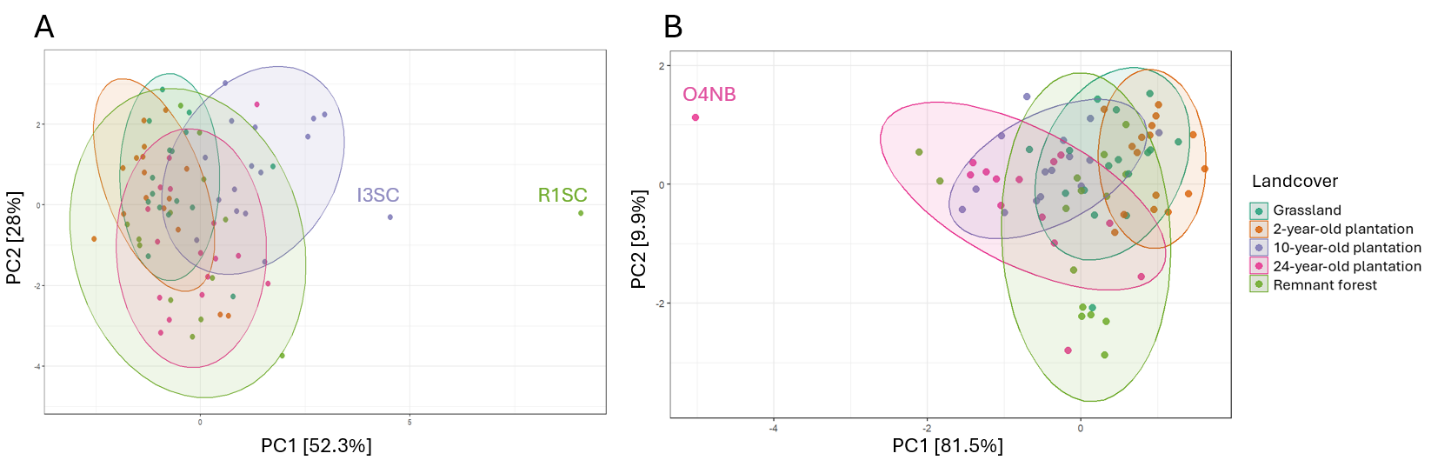
**Fig. S6** Rarefaction curve of bacteria.

**Fig. S7** Fungi (A) and bacteria (B) Principal Component Analysis (PCA) plots regarding functional groups for each landcover type. Acronyms highlight the origins of the outliers. Precisely, I3SC: 3^rd^ transect, 10-year-old plantation, southern side of the hill, and third plot; R1SC: 1^st^ transect, remnant forest, southern side of the hill, and third plot; O4NB: 4^th^ transect, 24-year-old plantation, northern side of the hill, and second plot.


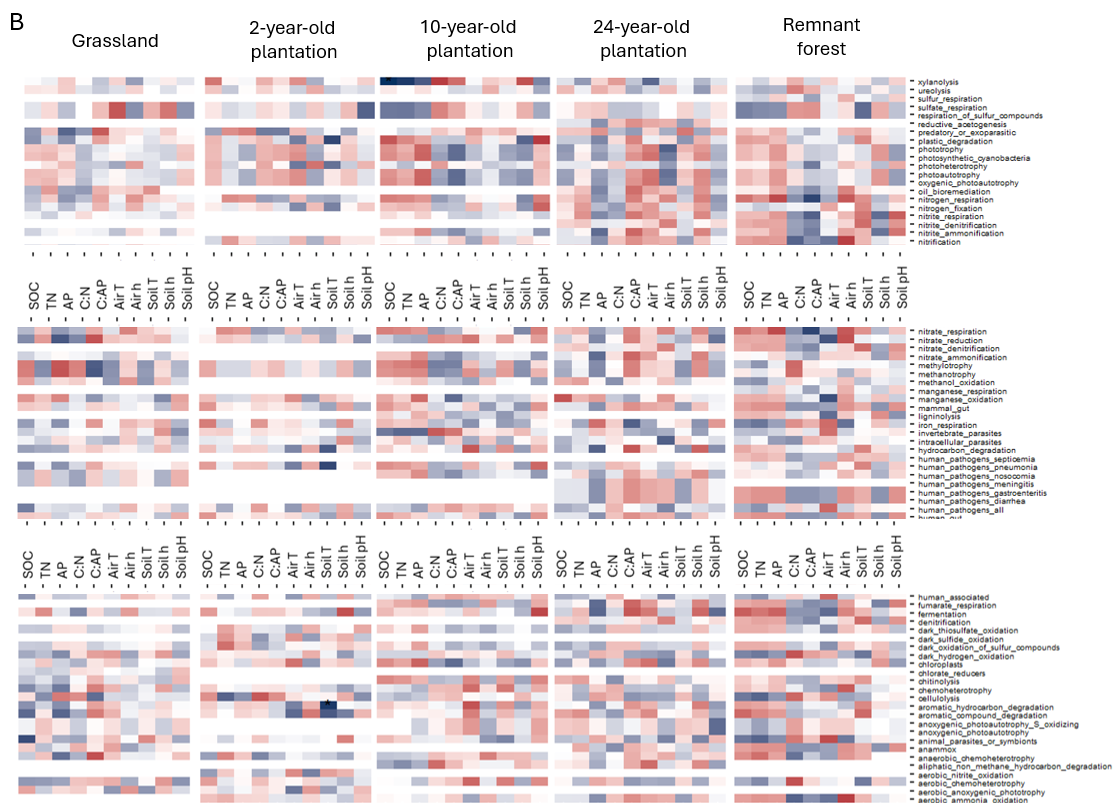

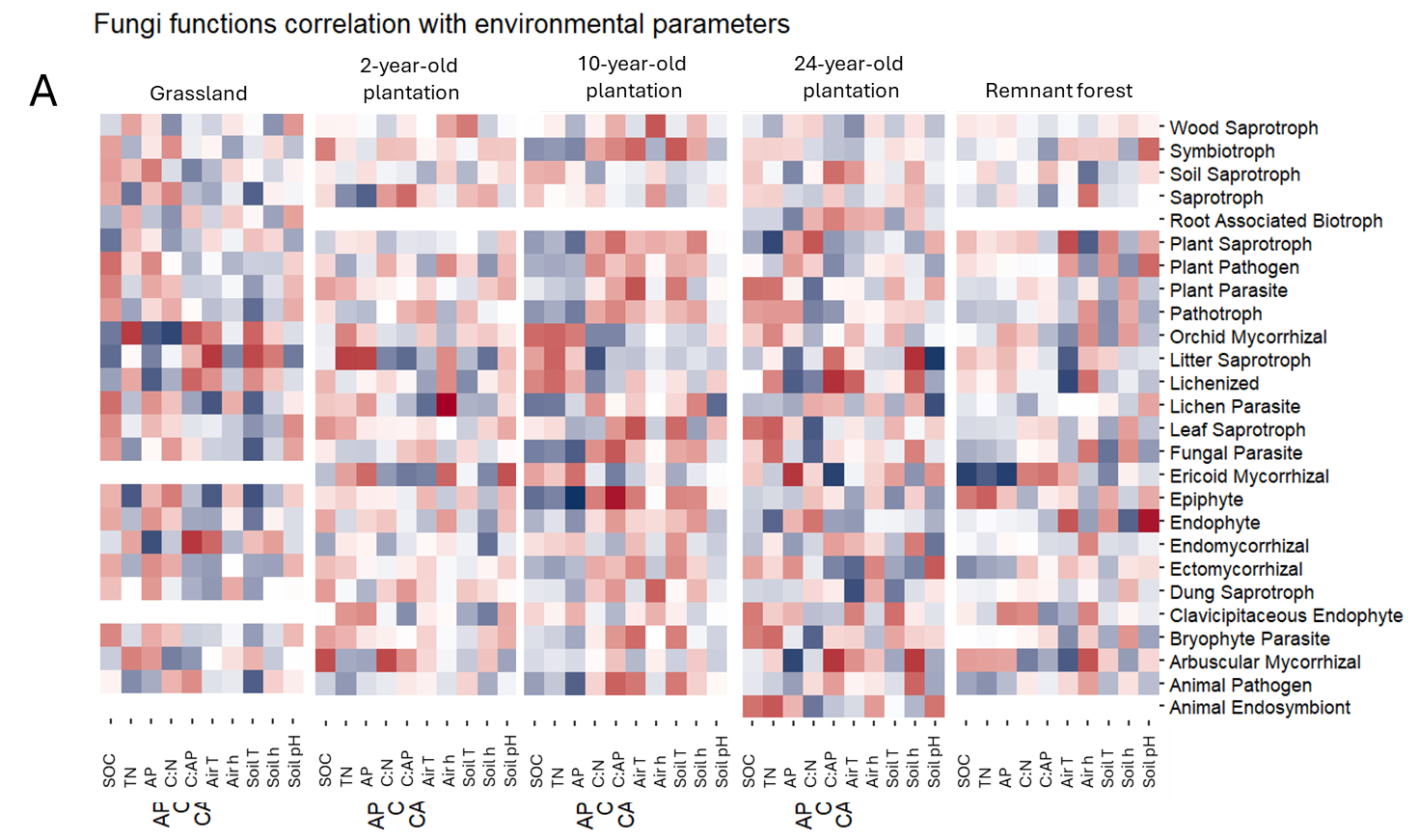
**Fig. S8** Correlation between the relative abundance of fungi guild (A) and bacteria functional groups (B) with environmental parameters. Red shades indicate positive correlation (through Spearman correlation coefficient), while blue gradations mark negative associations.


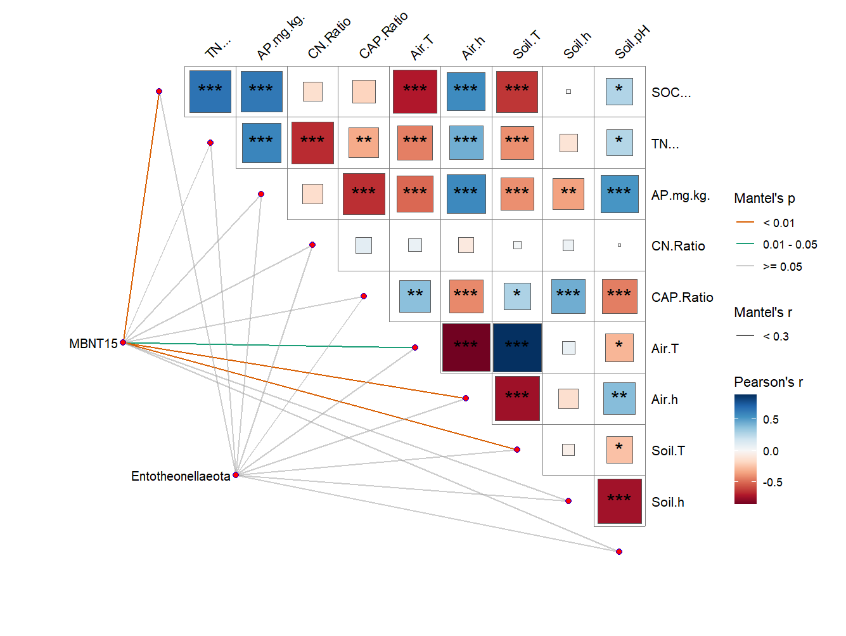


**Fig. S9** Correlation between MBNT15, Entotheonellaeota (as additional reference) bacteria phyla and environmental parameters.

**Tables**

**Table S1.** Abiotic parameters of the study sites used to assess the relationship between soil physicochemical properties and microbial communities. Average values for air and soil parameters are reported for each landcover type. Data deriving from Vivian et al., (2026).

|  | | Air | | Soil | | | | | |  |
| --- | --- | --- | --- | --- | --- | --- | --- | --- | --- | --- |
|  | **Elevation (m.a.s.l.)** | **Temperature (°C )** | **Moisture (%)** | **Temperature (°C )** | **Humidity (%)** | **pH** | **SOC (%)** | **Total Nitrogen (%)** | **Available Phosphorus (mg/kg)** | **C:N** |
| Grassland | 188.6±10.0A | 34.4±2.6 A | 65.6±5.7 C | 31.0±1.5 A | 55.8±17.8 A | 5.6±0.5 BC | 4.3±0.5 B | 0.2±0.1 C | 6.8±2.5 B | 40±35 A |
| 2-year-old plantation | 217.4±31.8 A | 35.1±2.2 A | 61.2±7.3 D | 30.5±1.3 A | 61.9±18.7 A | 5.4±0.4 C | 4.7±0.9 B | 0.3±0.0 B | 6±2.3 B | 18±7 B |
| 10-year-old plantation | 296.3±14.0 B | 30±1.2 B | 74.4±2.0 B | 27.4±0.7 B | 53.4±11.7 A | 6.2±0.4 A | 6.3±0.5 A | 0.4±0.1 A | 13.7±6.3 A | 16±2 B |
| 24-year-old plantation | 313.9±44.1 B | 29.1±1 BC | 78.3±6.4 AB | 26.8±1.0 BC | 64.9±12.7 A | 5.7±0.5 BC | 6.3±0.5 A | 0.4±0.1 A | 10.7±4.9 A | 22±24 B |
| Remnant forest | 405.9±82.4 C | 28.2±1.6 C | 81.1±6.4 A | 26.1±0.5 C | 56.5±14.8 A | 5.9±0.5 B | 6.6±1.6 A | 0.4±0.1 A | 13.8±7.4 A | 16±2 B |

**Table S2.** LefSe analysis for bacterial genera, with the landcover in which they are most abundant.

| **Taxa** | **Group** | **LDA** | **P.unadj** | **P.adj** | **Significance** |
| --- | --- | --- | --- | --- | --- |
| Edaphobacter | Grassland | 3.52 | 6.32E-06 | 0.00033 | *** |
| FCPS473 | Grassland | 3.47 | 1.38E-10 | 3.73E-08 | *** |
| Gemmata | Grassland | 3.30 | 0.000318 | 0.005252 | ** |
| Bacillus | Grassland | 3.29 | 0.000391 | 0.006092 | ** |
| Conexibacter | Grassland | 3.19 | 9.41E-05 | 0.002458 | ** |
| Pedomicrobium | Grassland | 3.13 | 3.37E-05 | 0.00105 | ** |
| Neobacillus | Grassland | 3.13 | 9.79E-06 | 0.000431 | *** |
| Anaeromyxobacter | Grassland | 3.12 | 4.40E-08 | 4.19E-06 | *** |
| Micromonospora | Grassland | 2.99 | 1.14E-08 | 1.54E-06 | *** |
| Dictyobacter | Grassland | 2.97 | 0.00074 | 0.009663 | ** |
| FFCH7168 | Grassland | 1.94 | 0.000383 | 0.006089 | ** |
| Geodermatophilus | Grassland | 1.94 | 0.000104 | 0.002635 | ** |
| Fimbriiglobus | Grassland | 2.81 | 0.000163 | 0.003665 | ** |
| Fictibacillus | Grassland | 2.66 | 7.60E-07 | 5.13E-05 | *** |
| Rhizobacter | Grassland | 2.54 | 1.01E-05 | 0.000431 | *** |
| Blastococcus | Grassland | 2.15 | 3.37E-05 | 0.00105 | ** |
| Kineosporia | Grassland | 2.14 | 0.000769 | 0.009729 | ** |
| HSB OF53-F07 | 2-year-old | 3.50 | 1.80E-05 | 0.000661 | *** |
| Ellin6067 | 2-year-old | 3.24 | 6.52E-06 | 0.00033 | *** |
| 1921-2 | 2-year-old | 3.22 | 1.33E-05 | 0.00054 | *** |
| Tautonia | 2-year-old | 3.13 | 4.66E-08 | 4.19E-06 | *** |
| Priestia | 2-year-old | 2.71 | 1.14E-10 | 3.73E-08 | *** |
| Domibacillus | 2-year-old | 2.34 | 0.000232 | 0.004373 | ** |
| Kinneretia | 2-year-old | 2.33 | 0.000286 | 0.004925 | ** |
| Chthonomonas | 2-year-old | 2.30 | 0.000654 | 0.00913 | ** |
| Phaselicystis | 2-year-old | 2.67 | 0.000168 | 0.003686 | ** |
| 1921-3 | 2-year-old | 2.54 | 0.000212 | 0.004284 | ** |
| Parapedobacter | 10-year-old | 1.70 | 0.000258 | 0.004749 | ** |
| Ellin516 | 10-year-old | 3.03 | 2.19E-07 | 1.62E-05 | *** |
| Crossiella | 10-year-old | 3.00 | 8.72E-05 | 0.002355 | ** |
| Terracidiphilus | 10-year-old | 3.22 | 2.12E-05 | 0.000748 | *** |
| Acidibacter | 10-year-old | 3.85 | 0.000652 | 0.00913 | ** |
| Acidiferrimicrobium | 10-year-old | 3.41 | 3.15E-05 | 0.00105 | ** |
| Silvibacterium | 10-year-old | 2.84 | 0.000222 | 0.004373 | ** |
| Catenulispora | 10-year-old | 2.62 | 7.57E-06 | 0.000361 | *** |
| Rickettsiella | 10-year-old | 2.61 | 0.000326 | 0.005289 | ** |
| Actinocatenispora | 10-year-old | 2.01 | 0.000513 | 0.007696 | ** |
| Hypericibacter | 24-year-old | 3.42 | 1.00E-07 | 8.10E-06 | *** |
| Nitrospira | 24-year-old | 3.19 | 2.80E-10 | 5.68E-08 | *** |
| Mycobacterium | 24-year-old | 3.35 | 0.000283 | 0.004925 | ** |
| Cupriavidus | 24-year-old | 2.94 | 1.24E-06 | 7.74E-05 | *** |
| Novosphingobium | 24-year-old | 2.79 | 2.51E-09 | 4.06E-07 | *** |
| GOUTA6 | 24-year-old | 2.26 | 7.35E-05 | 0.002054 | ** |
| Methylovirgula | 24-year-old | 2.55 | 4.18E-06 | 0.000242 | *** |
| Candidatus Soleaferrea | 24-year-old | 2.15 | 0.000589 | 0.008525 | ** |
| Pseudolabrys | Remnant | 3.71 | 5.15E-11 | 3.73E-08 | *** |
| mle1-7 | Remnant | 3.13 | 7.35E-05 | 0.002054 | ** |
| Steroidobacter | Remnant | 3.11 | 0.000132 | 0.003046 | ** |
| Terrimonas | Remnant | 3.11 | 0.000297 | 0.005018 | ** |
| SWB02 | Remnant | 3.11 | 7.32E-05 | 0.002054 | ** |
| Rhizobium | Remnant | 2.94 | 1.92E-08 | 2.23E-06 | *** |
| Ilumatobacter | Remnant | 2.72 | 0.00012 | 0.002934 | ** |
| Rubrivivax | Remnant | 2.69 | 0.000183 | 0.00391 | ** |
| Hassallia | Remnant | 2.62 | 0.000442 | 0.006761 | ** |
| CL500-29 marine group | Remnant | 2.52 | 0.000757 | 0.009729 | ** |
| Rurimicrobium | Remnant | 2.36 | 1.71E-05 | 0.000659 | *** |
| JTB255 marine benthic group | Remnant | 2.18 | 0.00058 | 0.008525 | ** |
| BD1-7 clade | Remnant | 2.17 | 0.000265 | 0.004777 | ** |
| Anaerocolumna | Remnant | 2.16 | 0.00023 | 0.004373 | ** |
| Candidatus Alysiosphaera | Remnant | 2.09 | 0.000701 | 0.009522 | ** |
| Adhaeribacter | Remnant | 2.07 | 0.00019 | 0.003954 | ** |
| Kineobactrum | Remnant | 2.05 | 0.000727 | 0.009656 | ** |
| Peredibacter | Remnant | 1.92 | 0.00013 | 0.003046 | ** |
| Solitalea | Remnant | 1.91 | 0.000705 | 0.009522 | ** |

**Table S3.** P-values of PERMANOVA results with the Jaccard distance metric.

| **Landcover** | **Fungi** | | | | | | **Bacteria** | | | | |
| --- | --- | --- | --- | --- | --- | --- | --- | --- | --- | --- | --- |
|  | **Grassland** | **2-year-old plantation** | **10-year-old plantation** | **24-year-old plantation** | | **Remnant forest** | **Grassland** | **2-year-old plantation** | **10-year-old plantation** | **24-year-old plantation** | **Remnant forest** |
| **Grassland** |  |  |  |  |  | |  |  |  |  |  |
| **2-year-old plantation** | 0.001 |  |  |  |  | | 0.001 |  |  |  |  |
| **10-year-old plantation** | 0.001 | 0.001 |  |  |  | | 0.001 | 0.001 |  |  |  |
| **24-year-old plantation** | 0.001 | 0.001 |  |  |  | | 0.001 | 0.001 | 0.001 |  |  |
| **Remnant forest** | 0.001 | 0.001 | 0.001 | 0.005 |  | | 0.001 | 0.001 | 0.001 | 0.007 |  |

**Table S4.** Fungal (below the diagonal) and bacterial (above the diagonal) PERMDISP analysis results (with permuted p-value).

|  |  | **Bacteria** | | | | |
| --- | --- | --- | --- | --- | --- | --- |
|  |  | **Grassland** | **2-year-old plantation** | **10-year-old plantation** | **24-year-old plantation** | **Remnant forest** |
| **Fungi** | **Grassland** |  | 0.203 | 0.761 | 0.279 | 0.001 |
|  | **2-year-old plantation** | 0.159 |  | 0.250 | 0.774 | 0.001 |
|  | **10-year-old plantation** | 0.001 | 0.037 |  | 0.356 | 0.001 |
|  | **24-year-old plantation** | 0.001 | 0.001 | 0.001 |  | 0.045 |
|  | **Remnant** | 0.001 | 0.001 | 0.001 | 0.962 |  |

**Table S5.** Unique ASV of fungi shared between landcover types, retrieved from Venn analysis.

| **Venn analysis Fungi** | | |
| --- | --- | --- |
| **Landcover** | **Number of ASVs** | **Total sequences** |
| 2-year-old plantation | 1383 | 113845 |
| 24-year-old plantation | 2443 | 189786 |
| Grassland | 1554 | 101622 |
| 10-year-old plantation | 1804 | 121893 |
| Remnant | 3080 | 236028 |
| 2-year-old plantation-24-year-old plantation | 14 | 3611 |
| 2-year-old plantation-Grassland | 550 | 341202 |
| 2-year-old plantation-10-year-old plantation | 43 | 12740 |
| 2-year-old plantation-Remnant | 13 | 1754 |
| 24-year-old plantation-Grassland | 13 | 3972 |
| 24-year-old plantation-10-year-old plantation | 245 | 117143 |
| 24-year-old plantation-Remnant | 342 | 109146 |
| Grassland-10-year-old plantation | 45 | 8459 |
| Grassland-Remnant | 27 | 3652 |
| 10-year-old plantation-Remnant | 104 | 26565 |
| 2-year-old plantation-24-year-old plantation-Grassland | 15 | 11495 |
| 2-year-old plantation-24-year-old plantation-10-year-old plantation | 4 | 2940 |
| 2-year-old plantation-24-year-old plantation-Remnant | 5 | 5455 |
| 2-year-old plantation-Grassland-10-year-old plantation | 58 | 125924 |
| 2-year-old plantation-Grassland-Remnant | 26 | 22272 |
| 2-year-old plantation-10-year-old plantation-Remnant | 5 | 1222 |
| 24-year-old plantation-Grassland-10-year-old plantation | 9 | 4456 |
| 24-year-old plantation-Grassland-Remnant | 8 | 2425 |
| 24-year-old plantation-10-year-old plantation-Remnant | 127 | 142899 |
| Grassland-10-year-old plantation-Remnant | 8 | 3109 |
| 2-year-old plantation-24-year-old plantation-Grassland-10-year-old plantation | 17 | 56700 |
| 2-year-old plantation-24-year-old plantation-Grassland-Remnant | 9 | 13176 |
| 2-year-old plantation-24-year-old plantation-10-year-old plantation-Remnant | 15 | 26548 |
| 2-year-old plantation-Grassland-10-year-old plantation-Remnant | 8 | 22319 |
| 24-year-old plantation-Grassland-10-year-old plantation-Remnant | 14 | 14641 |
| 2-year-old plantation-24-year-old plantation-Grassland-10-year-old plantation-Remnant | 18 | 87635 |

**Table S6.** Unique ASV of bacteria shared between landcover types, retrieved from Venn analysis.

| **Venn analysis Bacteria** | | |
| --- | --- | --- |
| **Landcover** | **Number of ASVs** | **Total sequences** |
| Remnant | 20847 | 158473 |
| 2-year-old plantation | 14217 | 126834 |
| 24-year-old plantation | 13176 | 89037 |
| Grassland | 13377 | 80181 |
| 10-year-old plantation | 14838 | 96066 |
| Remnant-2-year-old plantation | 169 | 7876 |
| Remnant-24-year-old plantation | 1330 | 109951 |
| Remnant-Grassland | 194 | 7471 |
| Remnant-10-year-old plantation | 364 | 21243 |
| 2-year-old plantation-24-year-old plantation | 100 | 3705 |
| 2-year-old plantation-Grassland | 1879 | 190238 |
| 2-year-old plantation-10-year-old plantation | 200 | 8513 |
| 24-year-old plantation-Grassland | 125 | 5348 |
| 24-year-old plantation-10-year-old plantation | 610 | 40964 |
| Grassland-10-year-old plantation | 178 | 7629 |
| Remnant-2-year-old plantation-24-year-old plantation | 60 | 5611 |
| Remnant-2-year-old plantation-Grassland | 165 | 24207 |
| Remnant-2-year-old plantation-10-year-old plantation | 59 | 5947 |
| Remnant-24-year-old plantation-Grassland | 116 | 14904 |
| Remnant-24-year-old plantation-10-year-old plantation | 677 | 181185 |
| Remnant-Grassland-10-year-old plantation | 65 | 7379 |
| 2-year-old plantation-24-year-old plantation-Grassland | 112 | 14343 |
| 2-year-old plantation-24-year-old plantation-10-year-old plantation | 60 | 5253 |
| 2-year-old plantation-Grassland-10-year-old plantation | 388 | 130898 |
| 24-year-old plantation-Grassland-10-year-old plantation | 83 | 7863 |
| Remnant-2-year-old plantation-24-year-old plantation-Grassland | 150 | 41422 |
| Remnant-2-year-old plantation-24-year-old plantation-10-year-old plantation | 127 | 45666 |
| Remnant-2-year-old plantation-Grassland-10-year-old plantation | 136 | 53431 |
| Remnant-24-year-old plantation-Grassland-10-year-old plantation | 220 | 95800 |
| 2-year-old plantation-24-year-old plantation-Grassland-10-year-old plantation | 140 | 68368 |
| Remnant-2-year-old plantation-24-year-old plantation-Grassland-10-year-old plantation | 620 | 1000533 |

**Table S7.** Significant correlations between fungal beta diversity and environmental variables.

| **Partial Mantel test for fungi communities in each landcover type** | | | |
| --- | --- | --- | --- |
| **Landcover type** | **Variables** | **Correlation coefficient (r)** | **p-value** |
| 2-year-old plantation | SOC (%) | 0.4 | 0.001 |
|  | C:N | 0.2 | 0.09 |
|  | C:AP | 0.4 | 0.02 |
| 24-year-old plantation | C:AP | 0.4 | 0.01 |
| Remnant forest | Air T (°C) | 0.3 | 0.01 |
|  | Air h (%) | 0.6 | 0.001 |

**Table S8.** Significant correlations between bacterial beta diversity and environmental variables.

| **Partial Mantel test for bacteria communities in each landcover type** | | | |
| --- | --- | --- | --- |
| **Land cover type** | **Variables** | **Correlation coefficient (r)** | **p-value** |
| Grassland | AP (mg/kg) | 0.4 | 0.04 |
|  | SOC (%) | 0.3 | 0.03 |
| 2-year-old plantation | SOC (%) | 0.4 | 0.001 |
|  | C:N | 0.3 | 0.004 |
|  | C:AP | 0.3 | 0.01 |
|  | Soil h (%) | 0.2 | 0.05 |
| 24-year-old plantation | C:AP | 0.4 | 0.02 |
| Remnant forest | TN (%) | 0.2 | 0.04 |
|  | Air T (°C) | 0.3 | 0.004 |
|  | Air h (%) | 0.5 | 0.003 |

**Table S9.** Relative abundance of fungi functional groups in each landcover type. Mean: mean relative abundance value. SD: Standard deviation.

| **Landcover** | **Taxa** | **Mean** | **SD** |
| --- | --- | --- | --- |
| Grassland | Saprotroph | 40.91 | 8.92 |
|  | Pathotroph | 22.52 | 8.29 |
|  | Fungal Parasite | 17.56 | 6.95 |
|  | Symbiotroph | 15.98 | 6.75 |
|  | Animal Pathogen | 15.90 | 4.84 |
|  | Wood Saprotroph | 9.40 | 6.49 |
|  | Dung Saprotroph | 6.40 | 5.46 |
|  | Ectomycorrhizal | 5.81 | 6.41 |
|  | Leaf Saprotroph | 4.86 | 6.44 |
|  | Plant Pathogen | 4.16 | 2.62 |
|  | Soil Saprotroph | 4.02 | 3.70 |
|  | Plant Parasite | 3.77 | 5.28 |
|  | Bryophyte Parasite | 3.75 | 5.28 |
|  | Endophyte | 3.42 | 2.48 |
|  | Orchid Mycorrhizal | 2.19 | 1.95 |
|  | Lichenized | 1.73 | 3.24 |
|  | Lichen Parasite | 1.24 | 1.82 |
|  | Plant Saprotroph | 0.94 | 0.83 |
|  | Arbuscular Mycorrhizal | 0.88 | 0.60 |
|  | Epiphyte | 0.36 | 1.09 |
|  | Litter Saprotroph | 0.11 | 0.15 |
|  | Endomycorrhizal | 0.05 | 0.13 |
|  | Root Associated Biotroph | 0.00 | 0.01 |
|  | Ericoid Mycorrhizal | 0.00 | 0.00 |
|  | Clavicipitaceous Endophyte | 0.00 | 0.00 |
|  | Animal Endosymbiont | 0.00 | 0.00 |
| 2-year-old plantation | Saprotroph | 45.54 | 9.12 |
|  | Pathotroph | 22.83 | 8.89 |
|  | Fungal Parasite | 20.28 | 8.71 |
|  | Animal Pathogen | 16.66 | 4.59 |
|  | Symbiotroph | 11.36 | 7.67 |
|  | Wood Saprotroph | 9.57 | 6.43 |
|  | Dung Saprotroph | 5.95 | 6.73 |
|  | Ectomycorrhizal | 5.42 | 6.99 |
|  | Leaf Saprotroph | 4.74 | 7.00 |
|  | Bryophyte Parasite | 4.67 | 6.98 |
|  | Plant Parasite | 4.64 | 6.99 |
|  | Soil Saprotroph | 3.44 | 3.88 |
|  | Endophyte | 2.41 | 1.50 |
|  | Plant Pathogen | 2.02 | 0.82 |
|  | Lichenized | 1.05 | 2.33 |
|  | Arbuscular Mycorrhizal | 0.84 | 0.44 |
|  | Plant Saprotroph | 0.82 | 1.21 |
|  | Orchid Mycorrhizal | 0.75 | 1.01 |
|  | Lichen Parasite | 0.41 | 0.35 |
|  | Epiphyte | 0.36 | 0.85 |
|  | Litter Saprotroph | 0.27 | 0.36 |
|  | Endomycorrhizal | 0.03 | 0.08 |
|  | Clavicipitaceous Endophyte | 0.01 | 0.03 |
|  | Ericoid Mycorrhizal | 0.01 | 0.02 |
|  | Root Associated Biotroph | 0.00 | 0.00 |
|  | Animal Endosymbiont | 0.00 | 0.00 |
| 10-year-old plantation | Saprotroph | 35.09 | 10.49 |
|  | Symbiotroph | 29.25 | 8.79 |
|  | Ectomycorrhizal | 19.50 | 8.24 |
|  | Pathotroph | 15.12 | 6.41 |
|  | Wood Saprotroph | 10.74 | 6.11 |
|  | Plant Pathogen | 8.44 | 4.09 |
|  | Endophyte | 7.87 | 3.07 |
|  | Fungal Parasite | 4.82 | 2.47 |
|  | Animal Pathogen | 4.41 | 2.77 |
|  | Soil Saprotroph | 2.97 | 1.58 |
|  | Dung Saprotroph | 2.18 | 1.69 |
|  | Epiphyte | 1.92 | 1.63 |
|  | Litter Saprotroph | 1.55 | 1.12 |
|  | Plant Saprotroph | 1.06 | 0.57 |
|  | Arbuscular Mycorrhizal | 0.86 | 0.87 |
|  | Lichen Parasite | 0.62 | 0.81 |
|  | Leaf Saprotroph | 0.51 | 0.70 |
|  | Bryophyte Parasite | 0.36 | 0.55 |
|  | Plant Parasite | 0.32 | 0.54 |
|  | Clavicipitaceous Endophyte | 0.31 | 0.55 |
|  | Ericoid Mycorrhizal | 0.20 | 0.59 |
|  | Orchid Mycorrhizal | 0.19 | 0.29 |
|  | Endomycorrhizal | 0.11 | 0.20 |
|  | Lichenized | 0.03 | 0.07 |
|  | Root Associated Biotroph | 0.00 | 0.00 |
|  | Animal Endosymbiont | 0.00 | 0.00 |
| 24-year-old plantation | Saprotroph | 51.92 | 12.87 |
|  | Symbiotroph | 23.72 | 7.81 |
|  | Pathotroph | 21.34 | 7.37 |
|  | Wood Saprotroph | 15.21 | 6.44 |
|  | Fungal Parasite | 11.97 | 6.90 |
|  | Ectomycorrhizal | 9.09 | 6.35 |
|  | Animal Pathogen | 8.17 | 5.68 |
|  | Endophyte | 8.01 | 5.75 |
|  | Plant Pathogen | 7.12 | 5.73 |
|  | Dung Saprotroph | 5.40 | 3.37 |
|  | Soil Saprotroph | 4.99 | 3.49 |
|  | Lichenized | 3.15 | 6.68 |
|  | Leaf Saprotroph | 2.82 | 3.99 |
|  | Plant Saprotroph | 2.09 | 2.56 |
|  | Bryophyte Parasite | 1.99 | 2.80 |
|  | Plant Parasite | 1.86 | 2.83 |
|  | Lichen Parasite | 1.37 | 1.53 |
|  | Arbuscular Mycorrhizal | 0.96 | 0.95 |
|  | Litter Saprotroph | 0.91 | 1.06 |
|  | Epiphyte | 0.68 | 0.70 |
|  | Orchid Mycorrhizal | 0.52 | 0.82 |
|  | Ericoid Mycorrhizal | 0.48 | 1.63 |
|  | Endomycorrhizal | 0.17 | 0.29 |
|  | Clavicipitaceous Endophyte | 0.07 | 0.15 |
|  | Animal Endosymbiont | 0.06 | 0.18 |
|  | Root Associated Biotroph | 0.01 | 0.03 |
| Remnant | Saprotroph | 48.05 | 13.93 |
|  | Pathotroph | 25.33 | 10.34 |
|  | Symbiotroph | 24.20 | 18.64 |
|  | Fungal Parasite | 19.53 | 12.49 |
|  | Ectomycorrhizal | 15.70 | 19.39 |
|  | Wood Saprotroph | 14.22 | 8.83 |
|  | Animal Pathogen | 11.24 | 6.89 |
|  | Dung Saprotroph | 7.75 | 8.79 |
|  | Bryophyte Parasite | 7.47 | 8.82 |
|  | Leaf Saprotroph | 7.15 | 8.92 |
|  | Plant Parasite | 6.77 | 8.85 |
|  | Endophyte | 4.60 | 3.62 |
|  | Plant Pathogen | 4.31 | 2.38 |
|  | Soil Saprotroph | 3.77 | 3.71 |
|  | Litter Saprotroph | 1.70 | 2.02 |
|  | Arbuscular Mycorrhizal | 1.24 | 1.77 |
|  | Lichenized | 0.99 | 1.19 |
|  | Plant Saprotroph | 0.89 | 1.61 |
|  | Orchid Mycorrhizal | 0.60 | 1.13 |
|  | Lichen Parasite | 0.56 | 0.54 |
|  | Epiphyte | 0.39 | 0.75 |
|  | Ericoid Mycorrhizal | 0.22 | 0.42 |
|  | Endomycorrhizal | 0.14 | 0.22 |
|  | Clavicipitaceous Endophyte | 0.11 | 0.20 |
|  | Root Associated Biotroph | 0.00 | 0.00 |
|  | Animal Endosymbiont | 0.00 | 0.00 |

**Table S10.** Relative abundance of bacteria functional groups in each landcover type. Mean: mean relative abundance value, on a scale from 0 to 1, which was converted to percentages in the main manuscript. SD: Standard deviation.

| **Landcover** | **Taxa** | **Mean** | **SD** |
| --- | --- | --- | --- |
| Grassland | chemoheterotrophy | 26.44 | 3.01 |
|  | aerobic chemoheterotrophy | 22.26 | 2.66 |
|  | dark hydrogen oxidation | 5.72 | 1.10 |
|  | anaerobic chemoheterotrophy | 4.17 | 1.80 |
|  | animal parasites or symbionts | 3.01 | 1.32 |
|  | cellulolysis | 1.48 | 0.72 |
|  | nitrate reduction | 0.59 | 0.51 |
|  | sulfate respiration | 0.43 | 0.20 |
|  | respiration of sulfur compounds | 0.43 | 0.20 |
|  | human associated | 0.35 | 0.56 |
|  | intracellular parasites | 0.34 | 0.25 |
|  | human pathogens all | 0.34 | 0.54 |
|  | fermentation | 0.32 | 0.64 |
|  | phototrophy | 0.20 | 0.15 |
|  | ureolysis | 0.19 | 0.10 |
|  | photoautotrophy | 0.19 | 0.15 |
|  | photosynthetic cyanobacteria | 0.19 | 0.16 |
|  | oxygenic photoautotrophy | 0.19 | 0.16 |
|  | aromatic compound degradation | 0.15 | 0.23 |
|  | chloroplasts | 0.13 | 0.15 |
|  | nitrogen fixation | 0.13 | 0.18 |
|  | iron respiration | 0.11 | 0.08 |
|  | human pathogens meningitis | 0.10 | 0.39 |
|  | xylanolysis | 0.09 | 0.19 |
|  | hydrocarbon degradation | 0.09 | 0.19 |
|  | aromatic hydrocarbon degradation | 0.08 | 0.19 |
|  | predatory or exoparasitic | 0.06 | 0.09 |
|  | human pathogens pneumonia | 0.06 | 0.14 |
|  | plastic degradation | 0.06 | 0.14 |
|  | nitrogen respiration | 0.06 | 0.12 |
|  | nitrate respiration | 0.06 | 0.12 |
|  | invertebrate parasites | 0.04 | 0.07 |
|  | dark thiosulfate oxidation | 0.03 | 0.09 |
|  | dark oxidation of sulfur compounds | 0.03 | 0.09 |
|  | nitrite respiration | 0.03 | 0.12 |
|  | nitrate ammonification | 0.03 | 0.12 |
|  | nitrite ammonification | 0.03 | 0.12 |
|  | human pathogens nosocomia | 0.02 | 0.09 |
|  | chitinolysis | 0.02 | 0.06 |
|  | manganese oxidation | 0.02 | 0.02 |
|  | human gut | 0.01 | 0.03 |
|  | mammal gut | 0.01 | 0.03 |
|  | methylotrophy | 0.01 | 0.01 |
|  | methanotrophy | 0.01 | 0.01 |
|  | oil bioremediation | 0.01 | 0.03 |
|  | photoheterotrophy | 0.01 | 0.01 |
|  | chlorate reducers | 0.00 | 0.01 |
|  | anoxygenic photoautotrophy S oxidizing | 0.00 | 0.00 |
|  | anoxygenic photoautotrophy | 0.00 | 0.00 |
|  | aerobic anoxygenic phototrophy | 0.00 | 0.01 |
|  | methanol oxidation | 0.00 | 0.00 |
|  | anammox | 0.00 | 0.00 |
|  | aerobic ammonia oxidation | 0.00 | 0.00 |
|  | aerobic nitrite oxidation | 0.00 | 0.00 |
|  | nitrification | 0.00 | 0.00 |
|  | sulfur respiration | 0.00 | 0.00 |
|  | nitrate denitrification | 0.00 | 0.00 |
|  | nitrite denitrification | 0.00 | 0.00 |
|  | denitrification | 0.00 | 0.00 |
|  | dark sulfide oxidation | 0.00 | 0.00 |
|  | manganese respiration | 0.00 | 0.00 |
|  | ligninolysis | 0.00 | 0.00 |
|  | human pathogens septicemia | 0.00 | 0.00 |
|  | human pathogens gastroenteritis | 0.00 | 0.00 |
|  | human pathogens diarrhea | 0.00 | 0.00 |
|  | aliphatic non methane hydrocarbon degradation | 0.00 | 0.00 |
|  | fumarate respiration | 0.00 | 0.00 |
|  | reductive acetogenesis | 0.00 | 0.00 |
| 2-year-old plantation | chemoheterotrophy | 23.29 | 1.99 |
|  | aerobic chemoheterotrophy | 18.85 | 2.04 |
|  | dark hydrogen oxidation | 5.66 | 1.16 |
|  | anaerobic chemoheterotrophy | 4.44 | 1.54 |
|  | cellulolysis | 1.62 | 1.13 |
|  | animal parasites or symbionts | 1.26 | 0.61 |
|  | sulfate respiration | 0.53 | 0.72 |
|  | respiration of sulfur compounds | 0.53 | 0.72 |
|  | phototrophy | 0.42 | 0.47 |
|  | photosynthetic cyanobacteria | 0.41 | 0.47 |
|  | oxygenic photoautotrophy | 0.41 | 0.47 |
|  | photoautotrophy | 0.41 | 0.47 |
|  | intracellular parasites | 0.38 | 0.16 |
|  | chloroplasts | 0.34 | 0.48 |
|  | nitrate reduction | 0.31 | 0.18 |
|  | iron respiration | 0.27 | 0.34 |
|  | fermentation | 0.21 | 0.14 |
|  | human associated | 0.17 | 0.11 |
|  | human pathogens all | 0.17 | 0.11 |
|  | ureolysis | 0.16 | 0.06 |
|  | nitrogen fixation | 0.07 | 0.10 |
|  | aromatic compound degradation | 0.06 | 0.05 |
|  | xylanolysis | 0.05 | 0.04 |
|  | hydrocarbon degradation | 0.05 | 0.06 |
|  | nitrification | 0.04 | 0.11 |
|  | aerobic nitrite oxidation | 0.03 | 0.11 |
|  | nitrate respiration | 0.03 | 0.06 |
|  | nitrogen respiration | 0.03 | 0.06 |
|  | invertebrate parasites | 0.03 | 0.04 |
|  | human pathogens pneumonia | 0.03 | 0.04 |
|  | methanotrophy | 0.02 | 0.04 |
|  | methylotrophy | 0.02 | 0.04 |
|  | aromatic hydrocarbon degradation | 0.02 | 0.03 |
|  | plastic degradation | 0.02 | 0.02 |
|  | photoheterotrophy | 0.02 | 0.03 |
|  | predatory or exoparasitic | 0.01 | 0.02 |
|  | dark oxidation of sulfur compounds | 0.01 | 0.03 |
|  | manganese oxidation | 0.01 | 0.01 |
|  | dark sulfide oxidation | 0.01 | 0.03 |
|  | aerobic anoxygenic phototrophy | 0.00 | 0.01 |
|  | dark thiosulfate oxidation | 0.00 | 0.01 |
|  | human gut | 0.00 | 0.01 |
|  | mammal gut | 0.00 | 0.01 |
|  | aerobic ammonia oxidation | 0.00 | 0.01 |
|  | methanol oxidation | 0.00 | 0.00 |
|  | sulfur respiration | 0.00 | 0.00 |
|  | anammox | 0.00 | 0.00 |
|  | nitrate denitrification | 0.00 | 0.00 |
|  | nitrite denitrification | 0.00 | 0.00 |
|  | denitrification | 0.00 | 0.00 |
|  | chitinolysis | 0.00 | 0.00 |
|  | nitrate ammonification | 0.00 | 0.00 |
|  | nitrite ammonification | 0.00 | 0.00 |
|  | nitrite respiration | 0.00 | 0.00 |
|  | manganese respiration | 0.00 | 0.00 |
|  | ligninolysis | 0.00 | 0.00 |
|  | human pathogens septicemia | 0.00 | 0.00 |
|  | human pathogens nosocomia | 0.00 | 0.00 |
|  | human pathogens meningitis | 0.00 | 0.00 |
|  | human pathogens gastroenteritis | 0.00 | 0.00 |
|  | human pathogens diarrhea | 0.00 | 0.00 |
|  | oil bioremediation | 0.00 | 0.00 |
|  | aliphatic non methane hydrocarbon degradation | 0.00 | 0.00 |
|  | fumarate respiration | 0.00 | 0.00 |
|  | chlorate reducers | 0.00 | 0.00 |
|  | anoxygenic photoautotrophy S oxidizing | 0.00 | 0.00 |
|  | anoxygenic photoautotrophy | 0.00 | 0.00 |
|  | reductive acetogenesis | 0.00 | 0.00 |
| 10-year-old plantation | chemoheterotrophy | 29.77 | 3.60 |
|  | aerobic chemoheterotrophy | 25.39 | 3.31 |
|  | dark hydrogen oxidation | 11.25 | 2.22 |
|  | anaerobic chemoheterotrophy | 4.37 | 1.06 |
|  | animal parasites or symbionts | 2.36 | 0.68 |
|  | cellulolysis | 1.56 | 0.66 |
|  | sulfate respiration | 0.84 | 0.34 |
|  | respiration of sulfur compounds | 0.84 | 0.34 |
|  | nitrate reduction | 0.70 | 0.49 |
|  | intracellular parasites | 0.40 | 0.18 |
|  | human associated | 0.34 | 0.11 |
|  | human pathogens all | 0.32 | 0.10 |
|  | iron respiration | 0.30 | 0.44 |
|  | fermentation | 0.20 | 0.19 |
|  | predatory or exoparasitic | 0.17 | 0.35 |
|  | phototrophy | 0.15 | 0.20 |
|  | photoautotrophy | 0.15 | 0.20 |
|  | photosynthetic cyanobacteria | 0.15 | 0.20 |
|  | oxygenic photoautotrophy | 0.15 | 0.20 |
|  | chloroplasts | 0.15 | 0.20 |
|  | ureolysis | 0.15 | 0.09 |
|  | invertebrate parasites | 0.11 | 0.08 |
|  | xylanolysis | 0.11 | 0.06 |
|  | aromatic compound degradation | 0.09 | 0.09 |
|  | nitrate respiration | 0.05 | 0.04 |
|  | nitrogen respiration | 0.05 | 0.04 |
|  | hydrocarbon degradation | 0.04 | 0.05 |
|  | aromatic hydrocarbon degradation | 0.03 | 0.05 |
|  | nitrogen fixation | 0.03 | 0.03 |
|  | human pathogens pneumonia | 0.02 | 0.03 |
|  | human gut | 0.01 | 0.04 |
|  | mammal gut | 0.01 | 0.04 |
|  | plastic degradation | 0.01 | 0.02 |
|  | aerobic ammonia oxidation | 0.01 | 0.03 |
|  | nitrification | 0.01 | 0.03 |
|  | manganese oxidation | 0.01 | 0.02 |
|  | methylotrophy | 0.01 | 0.01 |
|  | methanotrophy | 0.01 | 0.01 |
|  | chitinolysis | 0.00 | 0.01 |
|  | fumarate respiration | 0.00 | 0.01 |
|  | dark thiosulfate oxidation | 0.00 | 0.01 |
|  | dark oxidation of sulfur compounds | 0.00 | 0.01 |
|  | photoheterotrophy | 0.00 | 0.00 |
|  | nitrite respiration | 0.00 | 0.01 |
|  | aliphatic non methane hydrocarbon degradation | 0.00 | 0.01 |
|  | nitrate ammonification | 0.00 | 0.00 |
|  | nitrite ammonification | 0.00 | 0.00 |
|  | ligninolysis | 0.00 | 0.01 |
|  | human pathogens nosocomia | 0.00 | 0.00 |
|  | anoxygenic photoautotrophy S oxidizing | 0.00 | 0.00 |
|  | anoxygenic photoautotrophy | 0.00 | 0.00 |
|  | methanol oxidation | 0.00 | 0.00 |
|  | aerobic nitrite oxidation | 0.00 | 0.00 |
|  | sulfur respiration | 0.00 | 0.00 |
|  | anammox | 0.00 | 0.00 |
|  | nitrate denitrification | 0.00 | 0.00 |
|  | nitrite denitrification | 0.00 | 0.00 |
|  | denitrification | 0.00 | 0.00 |
|  | dark sulfide oxidation | 0.00 | 0.00 |
|  | manganese respiration | 0.00 | 0.00 |
|  | human pathogens septicemia | 0.00 | 0.00 |
|  | human pathogens meningitis | 0.00 | 0.00 |
|  | human pathogens gastroenteritis | 0.00 | 0.00 |
|  | human pathogens diarrhea | 0.00 | 0.00 |
|  | oil bioremediation | 0.00 | 0.00 |
|  | chlorate reducers | 0.00 | 0.00 |
|  | aerobic anoxygenic phototrophy | 0.00 | 0.00 |
|  | reductive acetogenesis | 0.00 | 0.00 |
| 24-year-old plantation | chemoheterotrophy | 0.01 | 0.01 |
|  | aerobic chemoheterotrophy | 0.00 | 0.01 |
|  | dark hydrogen oxidation | 0.01 | 0.02 |
|  | anaerobic chemoheterotrophy | 0.01 | 0.02 |
|  | animal parasites or symbionts | 0.00 | 0.00 |
|  | cellulolysis | 0.01 | 0.02 |
|  | sulfate respiration | 1.07 | 0.39 |
|  | respiration of sulfur compounds | 0.00 | 0.00 |
|  | nitrate reduction | 1.07 | 0.39 |
|  | intracellular parasites | 0.00 | 0.00 |
|  | phototrophy | 0.01 | 0.05 |
|  | photoautotrophy | 0.01 | 0.05 |
|  | photosynthetic cyanobacteria | 0.01 | 0.05 |
|  | oxygenic photoautotrophy | 0.00 | 0.00 |
|  | chloroplasts | 11.11 | 5.21 |
|  | human associated | 0.05 | 0.06 |
|  | human pathogens all | 0.02 | 0.05 |
|  | iron respiration | 0.03 | 0.09 |
|  | fermentation | 0.03 | 0.09 |
|  | invertebrate parasites | 1.96 | 1.39 |
|  | predatory or exoparasitic | 0.10 | 0.06 |
|  | ureolysis | 0.00 | 0.00 |
|  | xylanolysis | 0.01 | 0.02 |
|  | aromatic compound degradation | 0.01 | 0.02 |
|  | nitrogen respiration | 0.01 | 0.01 |
|  | nitrate respiration | 0.00 | 0.00 |
|  | nitrogen fixation | 0.00 | 0.00 |
|  | nitrite respiration | 0.27 | 0.41 |
|  | hydrocarbon degradation | 27.75 | 7.26 |
|  | nitrite ammonification | 0.20 | 0.24 |
|  | nitrate ammonification | 0.00 | 0.00 |
|  | aromatic hydrocarbon degradation | 0.01 | 0.01 |
|  | nitrate denitrification | 0.00 | 0.01 |
|  | nitrite denitrification | 0.00 | 0.01 |
|  | denitrification | 0.01 | 0.04 |
|  | human gut | 0.01 | 0.04 |
|  | mammal gut | 0.45 | 0.37 |
|  | manganese oxidation | 0.01 | 0.05 |
|  | fumarate respiration | 0.45 | 0.37 |
|  | human pathogens gastroenteritis | 0.01 | 0.05 |
|  | human pathogens diarrhea | 2.23 | 0.82 |
|  | oil bioremediation | 0.01 | 0.03 |
|  | methylotrophy | 0.01 | 0.03 |
|  | photoheterotrophy | 0.09 | 0.17 |
|  | aliphatic non methane hydrocarbon degradation | 0.01 | 0.03 |
|  | methanotrophy | 0.03 | 0.05 |
|  | dark thiosulfate oxidation | 0.31 | 0.28 |
|  | dark oxidation of sulfur compounds | 0.07 | 0.16 |
|  | anoxygenic photoautotrophy S oxidizing | 0.92 | 0.39 |
|  | anoxygenic photoautotrophy | 0.08 | 0.16 |
|  | human pathogens pneumonia | 0.01 | 0.04 |
|  | aerobic ammonia oxidation | 0.54 | 0.38 |
|  | nitrification | 0.00 | 0.00 |
|  | plastic degradation | 0.18 | 0.10 |
|  | human pathogens nosocomia | 0.48 | 0.98 |
|  | human pathogens meningitis | 0.48 | 0.98 |
|  | methanol oxidation | 0.01 | 0.02 |
|  | anammox | 0.01 | 0.02 |
|  | chitinolysis | 0.48 | 0.98 |
|  | reductive acetogenesis | 0.49 | 0.98 |
|  | aerobic nitrite oxidation | 0.00 | 0.00 |
|  | sulfur respiration | 0.01 | 0.02 |
|  | dark sulfide oxidation | 0.49 | 0.98 |
|  | manganese respiration | 0.01 | 0.01 |
|  | ligninolysis | 0.16 | 0.06 |
|  | human pathogens septicemia | 0.00 | 0.00 |
|  | chlorate reducers | 33.01 | 5.76 |
|  | aerobic anoxygenic phototrophy | 5.26 | 2.22 |
| Remnant | chemoheterotrophy | 29.87 | 3.98 |
|  | aerobic chemoheterotrophy | 23.47 | 5.00 |
|  | dark hydrogen oxidation | 8.20 | 1.93 |
|  | anaerobic chemoheterotrophy | 6.40 | 2.74 |
|  | animal parasites or symbionts | 1.99 | 1.45 |
|  | nitrate reduction | 0.84 | 0.37 |
|  | sulfate respiration | 0.84 | 0.25 |
|  | respiration of sulfur compounds | 0.84 | 0.25 |
|  | cellulolysis | 0.83 | 0.87 |
|  | fermentation | 0.32 | 0.22 |
|  | intracellular parasites | 0.28 | 0.07 |
|  | human pathogens all | 0.23 | 0.14 |
|  | human associated | 0.23 | 0.14 |
|  | iron respiration | 0.18 | 0.29 |
|  | phototrophy | 0.15 | 0.18 |
|  | chloroplasts | 0.14 | 0.17 |
|  | photosynthetic cyanobacteria | 0.14 | 0.17 |
|  | oxygenic photoautotrophy | 0.14 | 0.17 |
|  | photoautotrophy | 0.14 | 0.17 |
|  | ureolysis | 0.13 | 0.11 |
|  | aromatic compound degradation | 0.11 | 0.13 |
|  | nitrogen respiration | 0.08 | 0.09 |
|  | nitrate respiration | 0.08 | 0.09 |
|  | predatory or exoparasitic | 0.08 | 0.06 |
|  | xylanolysis | 0.06 | 0.05 |
|  | invertebrate parasites | 0.06 | 0.06 |
|  | hydrocarbon degradation | 0.04 | 0.07 |
|  | aromatic hydrocarbon degradation | 0.04 | 0.07 |
|  | plastic degradation | 0.04 | 0.07 |
|  | dark thiosulfate oxidation | 0.03 | 0.06 |
|  | dark oxidation of sulfur compounds | 0.03 | 0.06 |
|  | manganese oxidation | 0.03 | 0.06 |
|  | chitinolysis | 0.03 | 0.06 |
|  | nitrogen fixation | 0.03 | 0.04 |
|  | ligninolysis | 0.02 | 0.06 |
|  | human pathogens pneumonia | 0.02 | 0.04 |
|  | aerobic ammonia oxidation | 0.01 | 0.03 |
|  | nitrification | 0.01 | 0.03 |
|  | nitrite respiration | 0.01 | 0.02 |
|  | nitrite ammonification | 0.01 | 0.02 |
|  | human pathogens nosocomia | 0.01 | 0.02 |
|  | nitrate ammonification | 0.01 | 0.02 |
|  | oil bioremediation | 0.01 | 0.02 |
|  | methylotrophy | 0.01 | 0.01 |
|  | photoheterotrophy | 0.01 | 0.01 |
|  | methanotrophy | 0.00 | 0.01 |
|  | nitrate denitrification | 0.00 | 0.01 |
|  | nitrite denitrification | 0.00 | 0.01 |
|  | denitrification | 0.00 | 0.01 |
|  | methanol oxidation | 0.00 | 0.01 |
|  | fumarate respiration | 0.00 | 0.01 |
|  | human pathogens gastroenteritis | 0.00 | 0.01 |
|  | human pathogens diarrhea | 0.00 | 0.01 |
|  | human gut | 0.00 | 0.01 |
|  | mammal gut | 0.00 | 0.01 |
|  | sulfur respiration | 0.00 | 0.00 |
|  | anammox | 0.00 | 0.00 |
|  | manganese respiration | 0.00 | 0.00 |
|  | human pathogens septicemia | 0.00 | 0.00 |
|  | anoxygenic photoautotrophy S oxidizing | 0.00 | 0.00 |
|  | anoxygenic photoautotrophy | 0.00 | 0.00 |
|  | aerobic nitrite oxidation | 0.00 | 0.00 |
|  | dark sulfide oxidation | 0.00 | 0.00 |
|  | human pathogens meningitis | 0.00 | 0.00 |
|  | aliphatic non methane hydrocarbon degradation | 0.00 | 0.00 |
|  | chlorate reducers | 0.00 | 0.00 |
|  | aerobic anoxygenic phototrophy | 0.00 | 0.00 |
|  | reductive acetogenesis | 0.00 | 0.00 |

**Table S11.** Significance of difference in functional groups abundance of fungi communities, from Kruskal-Wallis and Dunn’s test. Differences of the taxa are limited to the landcover.

| **Taxa** | **Landcover** | **Significance** |
| --- | --- | --- |
| Pathotroph | Remnant | a |
|  | Grassland | ab |
|  | 2-year-old | ab |
|  | 24-year-old | ab |
|  | 10-year-old | b |
| Saprotroph | 24-year-old | a |
|  | Remnant | a |
|  | 2-year-old | ab |
|  | Grassland | ab |
|  | 10-year-old | b |
| Symbiotroph | 10-year-old | a |
|  | 24-year-old | ab |
|  | Remnant | ab |
|  | Grassland | bc |
|  | 2-year-old | c |
| Bryophyte Parasite | Remnant | a |
|  | Grassland | a |
|  | 2-year-old | a |
|  | 24-year-old | a |
|  | 10-year-old | b |
| Dung Saprotroph | Grassland | a |
|  | Remnant | a |
|  | 2-year-old | ab |
|  | 24-year-old | a |
|  | 10-year-old | b |
| Ectomycorrhizal | 10-year-old | a |
|  | Remnant | ab |
|  | 24-year-old | b |
|  | Grassland | b |
|  | 2-year-old | b |
| Fungal Parasite | 2-year-old | a |
|  | Remnant | a |
|  | Grassland | a |
|  | 24-year-old | a |
|  | 10-year-old | b |
| Leaf Saprotroph | Remnant | a |
|  | Grassland | a |
|  | 2-year-old | a |
|  | 24-year-old | ab |
|  | 10-year-old | b |
| Plant Parasite | Remnant | a |
|  | Grassland | a |
|  | 2-year-old | a |
|  | 24-year-old | ab |
|  | 10-year-old | b |
| Wood Saprotroph | 24-year-old | a |
|  | Remnant | a |
|  | 10-year-old | a |
|  | 2-year-old | a |
|  | Grassland | a |
| Animal Pathogen | 2-year-old | a |
|  | Grassland | a |
|  | Remnant | ab |
|  | 24-year-old | bc |
|  | 10-year-old | c |
| Endophyte | 10-year-old | a |
|  | 24-year-old | a |
|  | Remnant | ab |
|  | Grassland | b |
|  | 2-year-old | b |
| Plant Pathogen | 10-year-old | a |
|  | 24-year-old | ab |
|  | Remnant | abc |
|  | Grassland | bc |
|  | 2-year-old | c |
| Lichen Parasite | 24-year-old | a |
|  | Grassland | a |
|  | 2-year-old | a |
|  | Remnant | a |
|  | 10-year-old | a |
| Litter Saprotroph | 10-year-old | a |
|  | Remnant | a |
|  | 24-year-old | ab |
|  | 2-year-old | bc |
|  | Grassland | c |
| Soil Saprotroph | 24-year-old | a |
|  | 10-year-old | a |
|  | Grassland | a |
|  | Remnant | a |
|  | 2-year-old | a |
| Plant Saprotroph | 10-year-old | a |
|  | 24-year-old | a |
|  | Grassland | a |
|  | Remnant | a |
|  | 2-year-old | a |
| Epiphyte | 10-year-old | a |
|  | 24-year-old | ab |
|  | Remnant | bc |
|  | Grassland | c |
|  | 2-year-old | c |
| Lichenized | Remnant | a |
|  | Grassland | a |
|  | 2-year-old | ab |
|  | 24-year-old | ab |
|  | 10-year-old | b |
| Arbuscular Mycorrhizal | 24-year-old | a |
|  | Grassland | a |
|  | Remnant | a |
|  | 2-year-old | a |
|  | 10-year-old | a |
| Endomycorrhizal | 24-year-old | a |
|  | Remnant | a |
|  | Grassland | a |
|  | 10-year-old | a |
|  | 2-year-old | a |
| Ericoid Mycorrhizal | Grassland | a |
|  | Remnant | a |
|  | 10-year-old | a |
|  | 24-year-old | a |
|  | 2-year-old | a |
| Orchid Mycorrhizal | Grassland | a |
|  | Remnant | b |
|  | 2-year-old | b |
|  | 24-year-old | b |
|  | 10-year-old | b |
| Root Associated Biotroph | Grassland | a |
|  | Remnant | a |
|  | 10-year-old | a |
|  | 24-year-old | a |
|  | 2-year-old | a |
| Clavicipitaceous Endophyte | 10-year-old | a |
|  | Remnant | ab |
|  | Grassland | b |
|  | 24-year-old | ab |
|  | 2-year-old | b |
| Animal Endosymbiont | Grassland | a |
|  | Remnant | a |
|  | 10-year-old | a |
|  | 24-year-old | b |
|  | 2-year-old | a |

**Table S12.** Significance of difference in functional groups abundance of bacteria communities, from Kruskal-Wallis and Dunn’s test.

| **Taxa** | **Landcover** | **Significance** |
| --- | --- | --- |
| methanotrophy | 2-year-old | a |
|  | Grassland | a |
|  | Remnant | a |
|  | 10-year-old | a |
|  | 24-year-old | a |
| methanol oxidation | Grassland | a |
|  | Remnant | a |
|  | 10-year-old | a |
|  | 24-year-old | a |
|  | 2-year-old | a |
| methylotrophy | 2-year-old | a |
|  | Grassland | a |
|  | Remnant | a |
|  | 10-year-old | a |
|  | 24-year-old | a |
| aerobic ammonia oxidation | Grassland | a |
|  | Remnant | a |
|  | 10-year-old | a |
|  | 24-year-old | a |
|  | 2-year-old | a |
| aerobic nitrite oxidation | Grassland | a |
|  | Remnant | a |
|  | 10-year-old | a |
|  | 24-year-old | a |
|  | 2-year-old | a |
| nitrification | Grassland | a |
|  | Remnant | a |
|  | 10-year-old | a |
|  | 24-year-old | a |
|  | 2-year-old | a |
| sulfate respiration | 24-year-old | a |
|  | 10-year-old | a |
|  | Remnant | a |
|  | Grassland | b |
|  | 2-year-old | b |
| sulfur respiration | Grassland | a |
|  | Remnant | a |
|  | 10-year-old | a |
|  | 24-year-old | a |
|  | 2-year-old | a |
| respiration of sulfur compounds | 24-year-old | a |
|  | 10-year-old | a |
|  | Remnant | a |
|  | Grassland | b |
|  | 2-year-old | b |
| anammox | Grassland | a |
|  | Remnant | a |
|  | 10-year-old | a |
|  | 24-year-old | a |
|  | 2-year-old | a |
| nitrate denitrification | Grassland | a |
|  | Remnant | a |
|  | 10-year-old | a |
|  | 24-year-old | a |
|  | 2-year-old | a |
| nitrite denitrification | Grassland | a |
|  | Remnant | a |
|  | 10-year-old | a |
|  | 24-year-old | a |
|  | 2-year-old | a |
| denitrification | Grassland | a |
|  | Remnant | a |
|  | 10-year-old | a |
|  | 24-year-old | a |
|  | 2-year-old | a |
| chitinolysis | Grassland | a |
|  | Remnant | a |
|  | 10-year-old | a |
|  | 24-year-old | a |
|  | 2-year-old | a |
| dark hydrogen oxidation | 24-year-old | a |
|  | 10-year-old | a |
|  | Remnant | a |
|  | Grassland | b |
|  | 2-year-old | b |
| nitrogen fixation | Grassland | a |
|  | 24-year-old | ab |
|  | 2-year-old | ab |
|  | 10-year-old | b |
|  | Remnant | b |
| nitrate ammonification | Grassland | a |
|  | Remnant | a |
|  | 10-year-old | a |
|  | 24-year-old | a |
|  | 2-year-old | a |
| nitrite ammonification | Grassland | a |
|  | Remnant | a |
|  | 10-year-old | a |
|  | 24-year-old | a |
|  | 2-year-old | a |
| nitrite respiration | Grassland | a |
|  | Remnant | a |
|  | 10-year-old | a |
|  | 24-year-old | a |
|  | 2-year-old | a |
| cellulolysis | 24-year-old | a |
|  | Grassland | ab |
|  | 10-year-old | ab |
|  | 2-year-old | ab |
|  | Remnant | b |
| xylanolysis | 10-year-old | a |
|  | 24-year-old | a |
|  | Grassland | a |
|  | 2-year-old | a |
|  | Remnant | a |
| dark sulfide oxidation | Grassland | a |
|  | Remnant | a |
|  | 10-year-old | a |
|  | 24-year-old | a |
|  | 2-year-old | a |
| dark thiosulfate oxidation | Grassland | a |
|  | Remnant | a |
|  | 10-year-old | a |
|  | 24-year-old | a |
|  | 2-year-old | a |
| dark oxidation of sulfur compounds | Grassland | a |
|  | Remnant | a |
|  | 10-year-old | a |
|  | 24-year-old | a |
|  | 2-year-old | a |
| manganese oxidation | Remnant | a |
|  | 24-year-old | a |
|  | Grassland | a |
|  | 10-year-old | a |
|  | 2-year-old | a |
| manganese respiration | Grassland | a |
|  | Remnant | a |
|  | 10-year-old | a |
|  | 24-year-old | a |
|  | 2-year-old | a |
| ligninolysis | Grassland | a |
|  | Remnant | b |
|  | 10-year-old | a |
|  | 24-year-old | a |
|  | 2-year-old | a |
| fermentation | Remnant | a |
|  | 2-year-old | a |
|  | Grassland | a |
|  | 24-year-old | a |
|  | 10-year-old | a |
| aerobic chemoheterotrophy | 24-year-old | a |
|  | 10-year-old | a |
|  | Remnant | a |
|  | Grassland | a |
|  | 2-year-old | b |
| invertebrate parasites | 10-year-old | a |
|  | 24-year-old | ab |
|  | Remnant | abc |
|  | Grassland | bc |
|  | 2-year-old | c |
| human pathogens septicemia | Grassland | a |
|  | Remnant | a |
|  | 10-year-old | a |
|  | 24-year-old | a |
|  | 2-year-old | a |
| human pathogens pneumonia | Grassland | a |
|  | 2-year-old | a |
|  | Remnant | a |
|  | 10-year-old | a |
|  | 24-year-old | a |
| human pathogens nosocomia | Grassland | a |
|  | Remnant | a |
|  | 10-year-old | a |
|  | 24-year-old | a |
|  | 2-year-old | a |
| human pathogens meningitis | Grassland | a |
|  | Remnant | a |
|  | 10-year-old | a |
|  | 24-year-old | a |
|  | 2-year-old | a |
| human pathogens gastroenteritis | Grassland | a |
|  | Remnant | a |
|  | 10-year-old | a |
|  | 24-year-old | a |
|  | 2-year-old | a |
| human pathogens diarrhea | Grassland | a |
|  | Remnant | a |
|  | 10-year-old | a |
|  | 24-year-old | a |
|  | 2-year-old | a |
| human pathogens all | 10-year-old | a |
|  | 24-year-old | a |
|  | Remnant | ab |
|  | Grassland | ab |
|  | 2-year-old | b |
| human gut | Grassland | a |
|  | Remnant | a |
|  | 10-year-old | a |
|  | 24-year-old | a |
|  | 2-year-old | a |
| human associated | 10-year-old | a |
|  | 24-year-old | a |
|  | Remnant | ab |
|  | Grassland | ab |
|  | 2-year-old | b |
| mammal gut | Grassland | a |
|  | Remnant | a |
|  | 10-year-old | a |
|  | 24-year-old | a |
|  | 2-year-old | a |
| animal parasites or symbionts | Grassland | a |
|  | 24-year-old | ab |
|  | 10-year-old | a |
|  | Remnant | ab |
|  | 2-year-old | b |
| oil bioremediation | Grassland | a |
|  | Remnant | a |
|  | 10-year-old | a |
|  | 24-year-old | a |
|  | 2-year-old | a |
| aromatic hydrocarbon degradation | Grassland | a |
|  | 10-year-old | a |
|  | 2-year-old | a |
|  | Remnant | a |
|  | 24-year-old | a |
| aromatic compound degradation | Remnant | a |
|  | 10-year-old | a |
|  | Grassland | a |
|  | 2-year-old | a |
|  | 24-year-old | a |
| aliphatic non methane hydrocarbon degradation | Grassland | a |
|  | Remnant | a |
|  | 10-year-old | a |
|  | 24-year-old | a |
|  | 2-year-old | a |
| hydrocarbon degradation | Grassland | a |
|  | 2-year-old | a |
|  | Remnant | a |
|  | 10-year-old | a |
|  | 24-year-old | a |
| iron respiration | 10-year-old | a |
|  | 24-year-old | a |
|  | 2-year-old | a |
|  | Grassland | a |
|  | Remnant | a |
| nitrate respiration | Remnant | a |
|  | 10-year-old | a |
|  | Grassland | a |
|  | 2-year-old | a |
|  | 24-year-old | a |
| nitrate reduction | 24-year-old | a |
|  | Remnant | ab |
|  | 10-year-old | ab |
|  | Grassland | bc |
|  | 2-year-old | c |
| nitrogen respiration | Remnant | a |
|  | 10-year-old | a |
|  | Grassland | a |
|  | 2-year-old | a |
|  | 24-year-old | a |
| fumarate respiration | Grassland | ab |
|  | Remnant | ab |
|  | 10-year-old | a |
|  | 24-year-old | ab |
|  | 2-year-old | b |
| intracellular parasites | 24-year-old | a |
|  | 10-year-old | a |
|  | 2-year-old | a |
|  | Remnant | a |
|  | Grassland | a |
| chlorate reducers | Grassland | a |
|  | Remnant | a |
|  | 10-year-old | a |
|  | 24-year-old | a |
|  | 2-year-old | a |
| predatory or exoparasitic | 24-year-old | a |
|  | Remnant | ab |
|  | 10-year-old | ab |
|  | Grassland | bc |
|  | 2-year-old | c |
| chloroplasts | 2-year-old | a |
|  | Remnant | a |
|  | Grassland | a |
|  | 24-year-old | a |
|  | 10-year-old | a |
| photosynthetic cyanobacteria | 2-year-old | a |
|  | Grassland | a |
|  | Remnant | a |
|  | 24-year-old | a |
|  | 10-year-old | a |
| anoxygenic photoautotrophy S oxidizing | Grassland | a |
|  | Remnant | a |
|  | 10-year-old | a |
|  | 24-year-old | a |
|  | 2-year-old | a |
| anoxygenic photoautotrophy | Grassland | a |
|  | Remnant | a |
|  | 10-year-old | a |
|  | 24-year-old | a |
|  | 2-year-old | a |
| oxygenic photoautotrophy | 2-year-old | a |
|  | Grassland | a |
|  | Remnant | a |
|  | 24-year-old | a |
|  | 10-year-old | a |
| photoautotrophy | 2-year-old | a |
|  | Grassland | a |
|  | 24-year-old | a |
|  | Remnant | a |
|  | 10-year-old | a |
| aerobic anoxygenic phototrophy | Grassland | a |
|  | Remnant | a |
|  | 10-year-old | a |
|  | 24-year-old | a |
|  | 2-year-old | a |
| photoheterotrophy | Grassland | a |
|  | Remnant | a |
|  | 10-year-old | a |
|  | 24-year-old | a |
|  | 2-year-old | a |
| phototrophy | 2-year-old | a |
|  | Grassland | a |
|  | Remnant | a |
|  | 24-year-old | a |
|  | 10-year-old | a |
| plastic degradation | Grassland | a |
|  | Remnant | a |
|  | 10-year-old | a |
|  | 24-year-old | a |
|  | 2-year-old | a |
| ureolysis | Grassland | a |
|  | 2-year-old | a |
|  | 24-year-old | a |
|  | Remnant | a |
|  | 10-year-old | a |
| reductive acetogenesis | Grassland | a |
|  | Remnant | a |
|  | 10-year-old | a |
|  | 24-year-old | a |
|  | 2-year-old | a |
| chemoheterotrophy | 24-year-old | a |
|  | 10-year-old | ab |
|  | Remnant | ab |
|  | Grassland | bc |
|  | 2-year-old | c |
| anaerobic chemoheterotrophy | Remnant | a |
|  | 10-year-old | a |
|  | 24-year-old | a |
|  | Grassland | a |
|  | 2-year-old | a |

**Table S13.** Relative abundance of the most distinct functions of fungi outliers (in italics), in comparison to samples belonging to the same landcover type. S: Symbiotroph; E: Ectomycorrhizal; P: Pathotroph; Sa: Saprotroph; BP: Bryophyte parasite; DS: Dung saprotroph; AP: Animal pathogen.

| **Samples** | **S** | **E** | **Samples** | **P** | **Sa** | **S** | **BP** | **DS** | **E** |
| --- | --- | --- | --- | --- | --- | --- | --- | --- | --- |
| I1SA | 23.82 | 14.02 | R4NB | 19.39 | 58.1 | 13.49 | 1.13 | 1.77 | 2.79 |
| I1SB | 37.9 | 27.32 | R3SC | 18.68 | 31.68 | 14.46 | 8.98 | 8.54 | 11.02 |
| I1SC | 27.83 | 17.26 | R3SD | 24.44 | 37.76 | 18.18 | 11.52 | 11.97 | 12.07 |
| I1SD | 35.24 | 26.02 | R3SB | 17.61 | 46.19 | 28.46 | 1.61 | 2.24 | 12.07 |
| I2NA | 17.55 | 14.22 | R2NA | 16.56 | 50.09 | 21.09 | 0.58 | 1.59 | 1.81 |
| I2NB | 23.97 | 10.21 | R4NA | 27.56 | 62.72 | 18.25 | 6.76 | 7.5 | 12.33 |
| I2NC | 20.2 | 16.66 | R4NC | 27.51 | 53.63 | 10.59 | 3.15 | 3.58 | 3.89 |
| I2ND | 20.01 | 8.37 | R2NC | 48.12 | 55.28 | 36.41 | 34.02 | 33.56 | 33.53 |
| I3SA | 29.71 | 20.53 | R2NB | 24.53 | 53.01 | 8.07 | 8.24 | 1.41 | 1.77 |
| I3SB | 34.54 | 22.8 | R2ND | 29.77 | 62.75 | 25.44 | 13.82 | 16.94 | 16.72 |
| *I3SC* | *53.69* | *42.08* | R1SD | 41.56 | 61.98 | 22.92 | 8.87 | 9.68 | 16.15 |
| I3SD | 27.08 | 14.95 | *R1SC* | *11.14* | *17.4* | *83.4* | *0.02* | *1.29* | *76.04* |
| I4NA | 34.92 | 27.48 | R3SA | 15.06 | 29.42 | 13.9 | 1.03 | 3.11 | 2.55 |
| I4NB | 27.04 | 14.89 | R4ND | 32.63 | 52.68 | 24.18 | 4.91 | 5.35 | 17.11 |
| I4NC | 29.45 | 17.47 |  |  |  |  |  |  |  |
| I4ND | 25.05 | 17.76 |  |  |  |  |  |  |  |

**Table S14.** Relative abundance of the most distinct functions of bacteria outlier (in italics), in comparison to samples belonging to the same landcover type. DHO: Dark hydrogen oxidation; AC: Aerobic chemoheterotrophy; HP: Human pathogens; HA: Human associated; IP: Intracellular parasites; C: Chemoheterotrophy; AnC: Anaerobic chemoheterotrophy.

| **Samples** | **DHO** | **AC** | **HP** | **HA** | **IP** | **C** | **AnC** |
| --- | --- | --- | --- | --- | --- | --- | --- |
| O1SA | 10.12 | 25.37 | 0.21 | 0.21 | 0.23 | 31.39 | 6.01 |
| O1SB | 11.2 | 28.57 | 0.15 | 0.15 | 0.05 | 33.96 | 5.39 |
| O1SC | 11.85 | 30.11 | 0.91 | 0.91 | 0.4 | 34.02 | 3.91 |
| O1SD | 11.51 | 29.24 | 0.53 | 0.53 | 0.88 | 33.59 | 4.35 |
| O2NA | 5.29 | 23.13 | 0.3 | 0.31 | 0.28 | 33.26 | 10.13 |
| O2NC | 7.37 | 20.9 | 0.13 | 0.13 | 0.23 | 27.13 | 6.23 |
| O2ND | 11.03 | 24.93 | 0.29 | 0.29 | 0.41 | 29.21 | 4.28 |
| O3SA | 12.77 | 30.78 | 0.96 | 0.96 | 1.03 | 35.2 | 4.43 |
| O3SB | 11.38 | 27.27 | 0.31 | 0.31 | 0.57 | 32.1 | 4.83 |
| O3SC | 12.56 | 30.99 | 0.62 | 0.62 | 0.93 | 34.77 | 3.77 |
| O3SD | 9.18 | 24.42 | 0.22 | 0.22 | 0.18 | 31.2 | 6.78 |
| *O4NB* | *27.15* | *49.23* | *1.32* | *1.32* | *1.33* | *50.58* | *1.35* |
| O4NC | 8.48 | 25.67 | 0.21 | 0.21 | 0.79 | 29.22 | 3.55 |
| O4ND | 5.58 | 17.89 | 0.13 | 0.13 | 0.31 | 26.55 | 8.65 |

**References**

Vivian, J., Chazdon, R. L., Catling, A. A., Shapcott, A., Herbohn, J., & Lee, D. J. (2026). *Acacia mangium* monocultures can catalyse the recovery of the tree community and aboveground carbon stock in the Philippines. *Forest Ecology and Management*, *601*, 123357. <https://doi.org/10.1016/j.foreco.2025.123357>
